# Tunable Circularly Polarized Luminescence via Chirality Induction and Energy Transfer from Organic Films to Semiconductor Nanocrystals

**DOI:** 10.1021/acsnano.2c06623

**Published:** 2022-11-07

**Authors:** Sylwia Parzyszek, Jacopo Tessarolo, Adrián Pedrazo-Tardajos, Ana M. Ortuño, Maciej Bagiński, Sara Bals, Guido H. Clever, Wiktor Lewandowski

**Affiliations:** † Faculty of Chemistry, University of Warsaw, 1 Pasteur Street, 02-093 Warsaw, Poland; ‡ Faculty of Chemistry and Chemical Biology, TU Dortmund University, Otto-Hahn Straße 6, 44227 Dortmund, Germany; § Electron Microscopy for Materials Research, University of Antwerp, Groenenborgerlaan, 171, 2020 Antwerp, Belgium; ∥ NANOlab Center of Excellence, University of Antwerp, 2020 Antwerp, Belgium

**Keywords:** supramolecular chirality, aggregation-induced emission, chirality amplification, liquid crystals, semiconductor nanocrystals

## Abstract

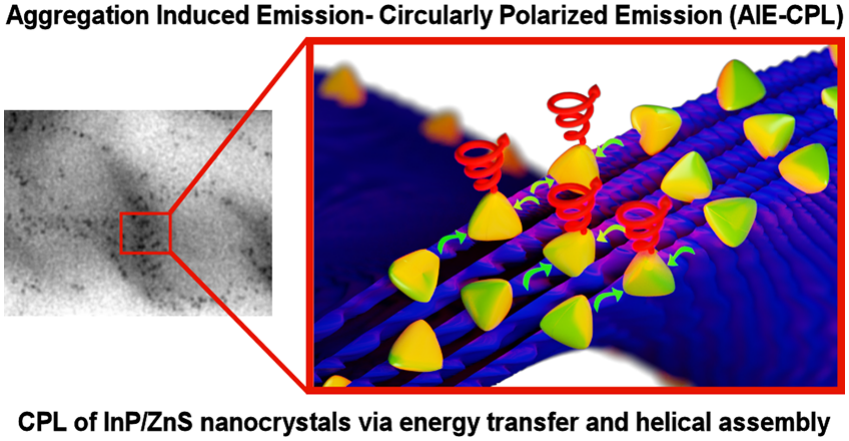

Circularly polarized luminescent (CPL) films with high dissymmetry factors hold great potential for optoelectronic applications. Herein, we propose a strategy for achieving strongly dissymetric CPL in nanocomposite films based on chirality induction and energy transfer to semiconductor nanocrystals. First, focusing on a purely organic system, aggregation-induced emission (AIE) and CPL activity of organic liquid crystals (LCs) forming helical nanofilaments was detected, featuring green emission with high dissymmetry factors *g*_lum_ ∼ 10^–2^. The handedness of helical filaments, and thus the sign of CPL, was controlled via minute amounts of a small chiral organic dopant. Second, nanocomposite films were fabricated by incorporating InP/ZnS semiconductor quantum dots (QDs) into the LC matrix, which induced the chiral assembly of QDs and endowed them with chiroptical properties. Due to the spectral matching of the components, energy transfer (ET) from LC to QDs was possible enabling a convenient way of tuning CPL wavelengths by varying the LC/QD ratio. As obtained, composite films exhibited absolute *g*_lum_ values up to ∼10^–2^ and thermally on/off switchable luminescence. Overall, we demonstrate the induction of chiroptical properties by the assembly of nonchiral building QDs on the chiral organic template and energy transfer from organic films to QDs, representing a simple and versatile approach to tune the CPL activity of organic materials.

## Introduction

Thin films generating circulalry polarized luminescence (CPL), that is exhibiting the differential emission intensity of right and left circularly polarized light, are fascinating from an intellectual, chirality-focused perspective while also providing clear solutions to some open problems in optoelectronic technologies. Increasing the bandwidth of data processing and storage,^1,2^ leveraging the contrast of light-emitting diodes and 3D displays,^3^ and developing anticounterfeiting types of tags with increased security^4,5^ are particularly interesting manifestations of the transformative potential of CPL-active films.^6−9^ Despite prominent successes in the areas of CPL, further efforts to maximize the applicative potential of CPL films are required, particularly developing strategies enabling enhancement, tuning, and active regulation of CPL activity in the solid state.^10^

Given the long history of organic luminogens, it is not surprising that CPL films are usually achieved by imparting chirality on conventional organic emitters.^6,11^ The early research mainly focused on the introduction of chiral units into metal complexes and small molecules. However, over the past decades increasing interest in CPL translated to rapid technological advancements. The variety of (i) materials, e.g., conjugated polymers,^12−14^ gels,^15−17^ liquid crystals,^18−22^ metal-organic,^23^ and covalent organic frameworks^24^ and cages,^25^ (ii) phenomena, e.g., thermally activated delayed fluorescence,^26,27^ room-temperature phosphorescence,^28−30^ and aggregation-induced emission (AIE),^31−33^ and (iii) approaches on imparting chirality, e.g., covalent and supramolecular,^15,34−38^ utilized within this field, is truly broad. Among the different approaches, achieving CPL in multicomponent systems exhibiting simultaneous chirality induction (induction of chiroptical properties of an achiral component) *and* energy transfer between constituents^15,19,39−44^ has been very recently shown to be particularly attractive. Such sophisticated systems enable advanced regulation,^39^ drastic amplification,^13^ and dynamic tunability^15,45,46^ of CPL fueling future research toward this way. Nevertheless, these systems are almost exclusively limited to the organic realm, not benefiting from the transformative potential of semiconductor nanocrystals (NCs) including QDs which offer high chemical stability, narrow and size-tunable emission, and high luminescence quantum yields. Additionally, incorporating QDs within multicomponent systems as chirality/energy acceptors could advance the CPL regulation, offering a tool for future optoelectronic technologies.

Until now, imparting chirality into inorganic NCs has been achieved through three major strategies: (i) preparing NCs showing intrinsic chirality, (ii) attaching chiral ligands to NC surfaces, and (iii) chiral assembly of single^47−51^ or multicomponent NC systems. The multicomponent assembly based approach relies on similar concepts as purely organic systems. In this approach, organic gelators,^52−54^ polymers,^55^ and liquid crystals^19,56−58^ have been explored as chiral hosts for achiral semiconductor NCs,^49^ either serving as a selective chiroptical filter or directing the chiral assembly of NCs. Although this approach provided access to full color/white-light CPL,^16,17,53^ switchable, and flexible CPL-active films,^55^ achieving multicomponent NC systems which exhibit induced chiroptical properties *and* energy transfer from the template to NCs remains elusive. Given that ET from organic luminophores to QDs is rare,^59−61^ this task is challenging, although worth the effort.^19,62^

Here, we develop multicomponent systems comprising an organic matrix and QDs which exhibit induced CPL properties through assembly and energy transfer mechanisms. In our work, we have chosen to use a liquid crystalline matrix that forms helically twisted fibers upon cooling low-molecular-weight organic molecules below the phase transition temperature.^63^ The handedness of the fibers is controlled via chirality amplification from minute amounts of a chiral dopant. Chirality is then imparted into InP/ZnS QDs that we designed to efficiently mix with the organic material at an elevated temperature and arrange in a helical geometry on twisted filaments upon phase transition. Interestingly, we show that molecules forming helical nanofilaments exhibit aggregation-induced CPL activity. By matching the matrix emission with InP/ZnS QDs absorption and benefiting from the distribution of QDs within the matrix, ET from matrix to QDs is achieved resulting in CPL activity of QDs. The full potential of the system is revealed by showing CPL tunability dependent on the matrix/QD ratio and temperature. Ultimately, our work leads to multicomponent CPL films that allow for convenient control over the sign, intensity, and wavelength of the CPL effect, benefiting from simultaneous chirality induction and energy transfer from an organic matrix to inorganic QDs.

## Results and Discussion

**Figure 1:**
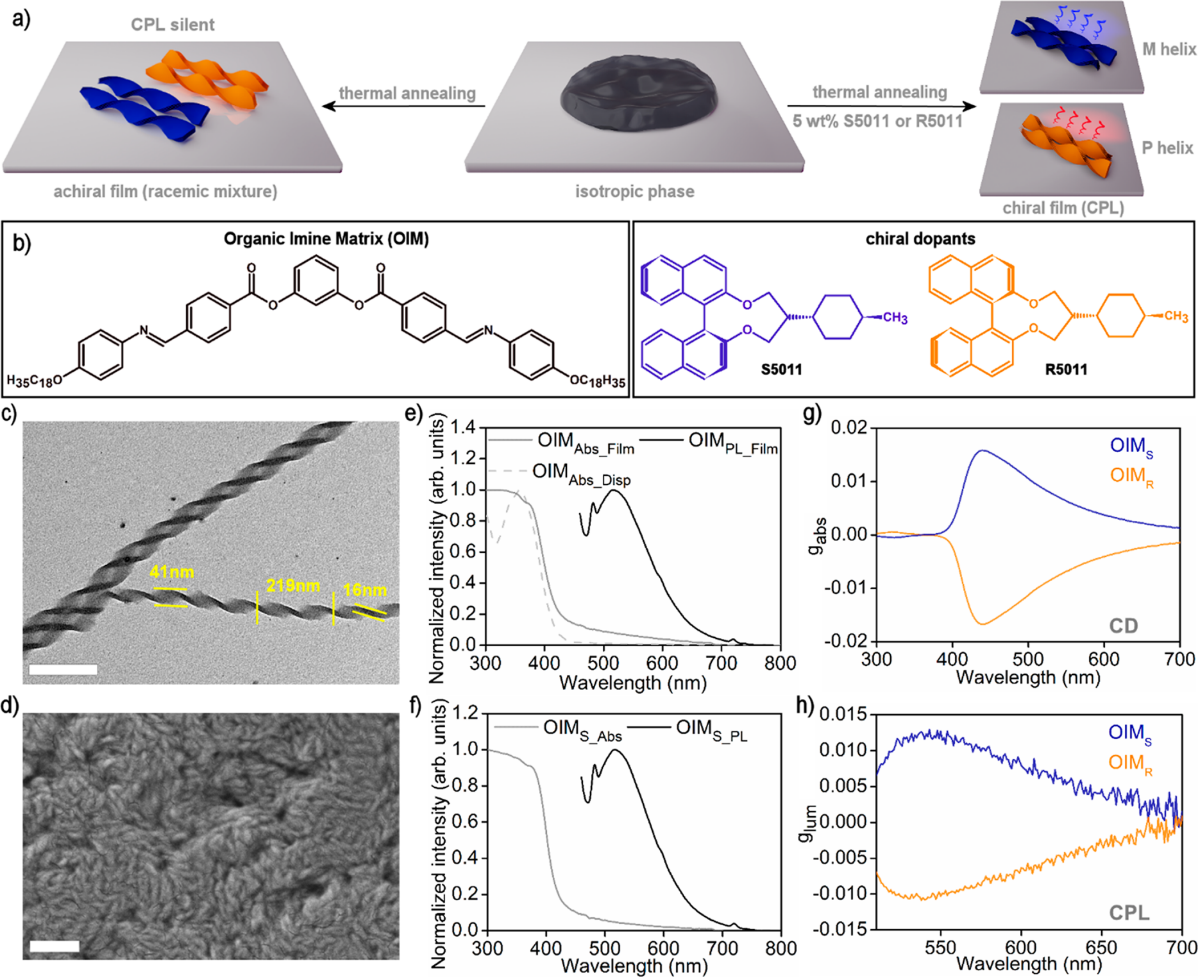
Structural and functional analysis of emissive organic films based on an organic-imine matrix (OIM). (a) Scheme of helical nanofilament formation by OIM on cooling from the isotropic melt. A conglomerate of domains of the opposite handedness is formed without a chiral dopant (on the left), while an excess of single-handed domains is formed in the presence of a chiral dopant (on the right). Samples doped with the chiral dopant exhibit aggregation-induced CPL. (b) Chemical structure of organic compounds used in the study: liquid crystal forming helical nanofilaments (OIM) and chiral dopants directing handedness of helical nanofilaments (R5011 and S5011). (c) Bright-field transmission electron microscopy (BF TEM) micrograph of a heat-annealed OIM which forms a splitting bundle of helices. The pitch of helices is 219 ± 5 nm, the width of the filament is 41 ± 1 nm, and the thickness is 16 ± 1 nm. (d) SEM micrograph of an OIM film. (e) Extinction (gray solid line) and photoluminescence (PL,black) normalized spectra of OIM in solid-state and THF solution (gray, dotted line). In the solution emission from OIM was not detected. (f) Extinction (gray) and PL (black) normalized spectra of OIM doped with 5 wt % chiral dopant. The addition of the dopant does not affect the position of PL spectra. (g, h) Circular dichroism and circularly polarized luminescence dissymmetry factors *g*_abs_ and *g*_lum_ of OIM films doped with R5011 or S5011, respectively. The ellipticity spectra are provided in Figures S17 and S18.

To prepare CPL-active, multicomponent films, we decided to use a thermotropic liquid crystal, an organic-imine matrix (OIM, Figure 1a,b) which comprises five aromatic rings connected via ester and imine moieties, constituting a stiff core of the molecule; the core is functionalized with two oleyl chains at opposite ends (Supplementary Notes 1, 2, 3). Previously, the OIM was shown to act as an efficient template to induce a double-helical arrangement of Au NPs, providing access to films exhibiting chiral plasmonic absorption.^64^ The driving mechanism is that upon cooling in the film state, the molecular architecture of the OIM supports the formation of layered assemblies. These layers twist into helical nanofilaments forming the helical nanofilament phase (HNF, called also the B4 phase) due to an internal torsion. Filaments grow dendritically from crystallization points, preserving their handedness and leading to the formation of sub-mm, chiral domains of both handedness; thus macroscopically the film is achiral. As will be discussed later, to achieve chiroptical properties (symmetry breaking) at the macroscopic level, a chiral, molecular dopant is required (Figure 1b).

The bright-field transmission electron microscopy (BF-TEM) micrograph of a drop-casted and heat-annealed, that is, heated to the isotropic phase and cooled to form HNF, OIM film is shown in Figure 1c (see also Supplementary Note 4). In thin areas, individual helical nanofilaments can be observed, whereas packing of the helices into dense structures leads to a periodic pattern of brighter and darker strips in thicker areas (Figure S13). The helical pitch of nanofilaments is 219 ± 5 nm, the width of the filament is 41 ± 1 nm, and the thickness is 16 ± 1 nm. To probe OIM’s morphology in films thicker than those probed via BF TEM, a liquid crystalline cell with a 3 μm gap and homogeneously orienting layers was filled with OIM, heat annealed, and frozen in liquid nitrogen. The film was accessed by separating glass substrates. Scanning electron microscopy (SEM) revealed that OIM film comprises twisted, elongated structures resembling those observed in BF TEM, confirming the three-dimensional, helical morphology of OIM (Figure 1d).

In the next step, the optical absorption and emission of OIM were examined in solution and solid-state (Figure 1e). Strong absorption bands in the UV region, characteristic of organic compounds, were revealed both in solution (λ_max_ ∼ 359 nm) and in solid-state measurements (λ_max_ ∼ 374 nm). In contrast, the emission of OIM varied between solution and solid state. Namely, solid-state assemblies exhibited PL centered at ∼510 nm, when excited with 440 nm light, while under the same conditions PL of the OIM solution was not detected, indicating that the OIM exhibits AIE. We decided to confirm that AIE reported for compounds forming HNF is not a phenomenon limited only to the studied compound. Indeed, AIE was detected in a film of another representative of the HNF family of LCs (Figure S16).

To achieve chiroptical properties at the macroscopic level, we decided to control the handedness of OIM’s helical nanofilaments by chirality amplification from low molecular weight chiral dopants (Supplementary Note 1). Films doped with 5 wt % 1,1′-bi-2-naphthol derivatives (either R5011 or S5011, referred to as OIM_R_ and OIM_S_ samples, respectively, Figure 1b; we will use OIM_R/S_ notation when referring to both homochiral samples) were prepared by drop casting and heat annealing. R5011 and S5011 dopants were previously shown to efficiently induce the chirality of polymer and LC films.^65,66^ Importantly, in comparison to previous works on dopants with asymmetric carbon, maximum absorption dissymmetry factors, *g*_abs_ values, were observed at lower concentration of dopants. Lower concentration translates to limited birefringence of the sample coming from the phase-segregated chiral dopant, which could potentially affect the measured circular dichroism (CD) spectra and CPL.^34,66^ The absorption (Abs) and photoluminescence (PL) spectra of OIM_R/S_ films revealed Abs and PL bands almost identical to those of pure OIM films (Figure 1f). These results attested that chiral dopants do not affect the Abs properties of OIM when using unpolarized light and do not contribute to PL. To test if OIM_R_ and OIM_S_ films exhibited chiroptical properties at the macroscopic level, they were further examined using CD and CPL spectroscopies in the transmission mode. In both cases, spectra were collected for four in-plane rotations (0, 90, 180, 270°) and after flipping the sample, to exclude contributions from linear dichroism and birefringence as well as circular birefringence. We observed nearly symmetrical, mirror signals confirming chiral absorption (Figure 1g, Figure S17) and chiral photoluminescence (Figure 1h, Figure S18) phenomena. CD bands with maxima at ∼437 nm and a maximal absolute value of *g*-factor (|*g*_abs_|) of up to 0.017 were recorded. Based on the previously reported, correlated SEM and CD measurements, we can conclude that the positive CD signal of OIM_S_ can be ascribed to the formation of left handed helical nanofilaments. The absolute value of luminescence dissymmetryfactor |*g*_lum_| reached the value of 0.013 (Tables S1 and S2). The acquired |*g*_lum_| values are comparable with other imine-based systems exhibiting AIE-CPL.^67^ With this part of the research we unequivocally confirmed the ability of OIM to form films with CPL activity in the green, unlocked by chirality amplification from small molecule dopant and AIE phenomena.

To regulate the CPL properties of the OIM_R/S_ films, we aimed at multicomponent system formation, namely, doping OIM_R/S_ with QDs. We chose InP/ZnS QDs as they exhibit a high quantum yield of PL in the vis region and they are considered an environmentally friendly alternative to cadmium-based nanocrystals.^68^ Moreover, these QDs are highly stable at elevated temperatures which is an essential feature from the perspective of OIM-based composite preparation.

To ensure an efficient *energy transfer* from OIM to InP/ZnS QDs, we regulated their optical properties through the control of ZnS shell thickness. Toward this aim, hot injection synthesis of InP QDs was followed by the gradual addition of sulfur and zinc precursors to coat InP nanocrystals with two layers of a ZnS shell, following the protocol of Tessier et al.^69^ We expected that Abs maxima of these QDs should be placed at ∼550 nm, overlapping with OIM’s emission.

**Figure 2:**
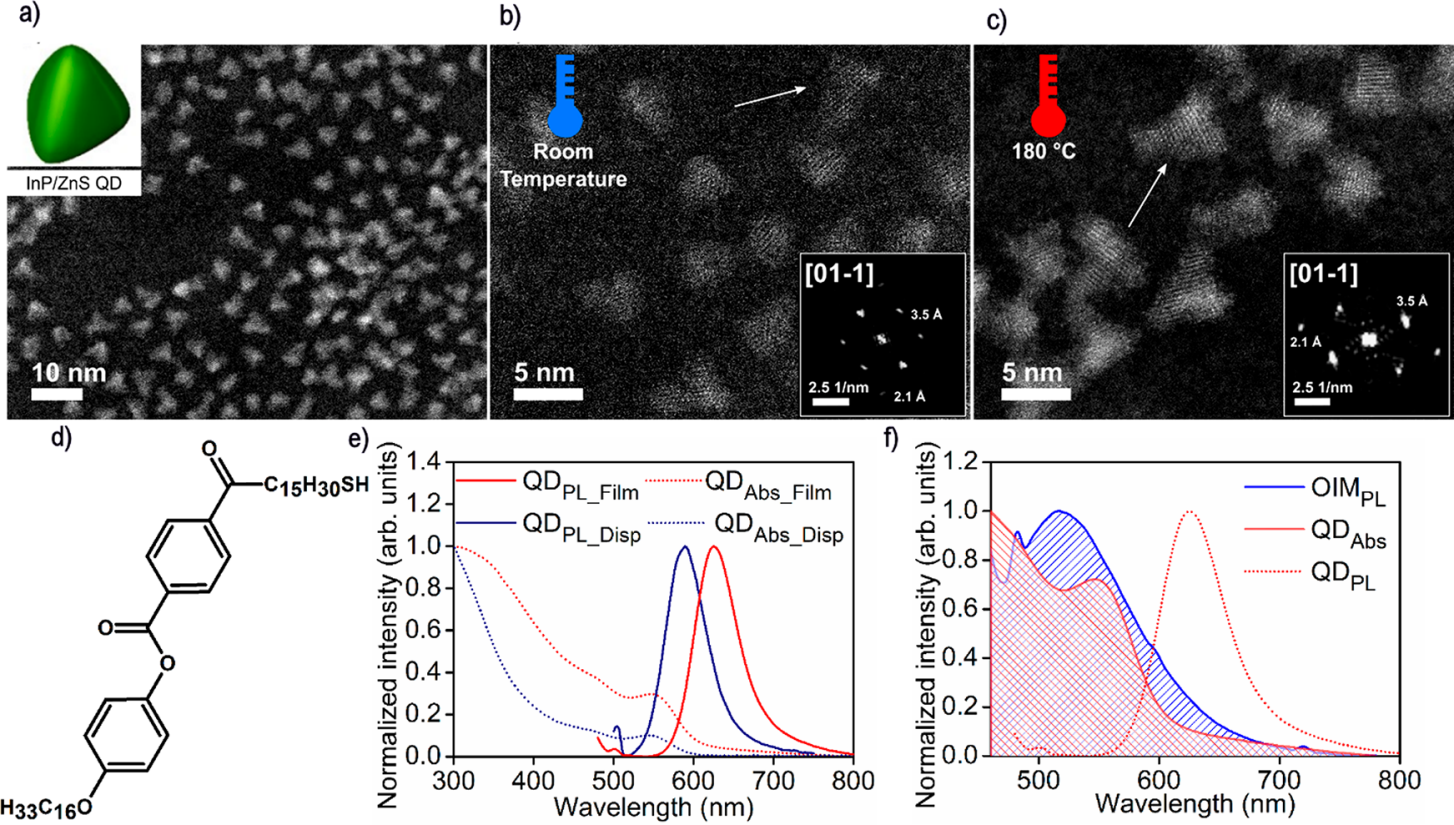
Structural and functional analysis of InP/ZnS quantum dots (QDs). (a) High-angle annular dark-field scanning transmission electron microscopy (HAADF-STEM) image of QDs suggests a tetrahedral shape of nanocrystals as presented by the model in the inset. (b) HAADF-STEM image of QDs acquired for InP/ZnS QDs at an ambient temperature. Insets show a fast Fourier transform (FFT) of QDs’ crystal lattice revealing a [01–1] zone axis. (c) HAADF-STEM image of QDs acquired for InP/ZnS QDs at an elevated temperature, confirming thermal stability of QDs. Insets show FFT of QDs’ crystal lattice revealing the same [01–1] zone axis as in the particle shown in panel b. (d) Chemical structure of a liquid crystalline-like ligand attached to the QDs surface in a ligand exchange process to ensure chemical compatibility of QDs with OIM. (e) Normalized Abs and PL spectra of QDs in dispersion and solid state. (f) Overlap of OIM emission with QDs absorption, important in the context of energy transfer between OIM and QDs.

To ensure an efficient *chirality induction* (Supplementary Note 5) to InP/ZnS QDs by the templated assembly on OIM_R/S_, the inorganic nanoparticles should be well distributed within the host matrix, adopting a helical morphology guided by the helical nanofilaments. Thus, InP/ZnS QDs should be chemically compatible with the OIM matrix, which we aimed to ensure by covering QDs with a mixed monolayer of dodecane- and LC-like ligands (Figure 2d and Figures S1–S4).^63,70,71^

HAADF-STEM (high-angle annular dark-field scanning transmission electron microscopy) analysis of obtained InP/ZnS QDs revealed the formation of 3.5 ± 0.4 nm nanocrystals likely with a tetrahedral shape (Figure 2a). The morphology and crystal structures of particles were highly stable against heating to 180 °C, as evidenced by high-resolution HAADF-STEM imaging of QDs at ambient and elevated temperatures (Figure 2b and Figure 2c respectively). In both cases (Figure 2b,c) the reflections correspond to a lattice spacing of ∼2.1 Å and ∼3.46 Å, corresponding to the crystal planes (220) and (111) respectively. Thus, we expected that patterns are consistent with the cubic zinc blende phases of InP/ZnS. The heat annealing procedure of multicomponent samples should not affect these QDs, since the nanocrystals remain single crystals with unchanged morphology. In dispersion, these nanocrystals exhibited Abs and PL bands centered at ∼545 and ∼589 nm, respectively (Figure 2d). In the film state both Abs and PL bands were red-shifted to ∼552 and ∼625 nm, respectively, attesting coupling between QDs in close-packed assemblies.^72^

Regarding the tentative resonant energy transfer phenomena, in which OIM would play the role of energy donor while QDs would play an energy acceptor, it can be noted that the extinction band of QDs overlaps the PL of OIM. For this donor–acceptor system, the spectral overlap function *J* was calculated (Figure S21). The value of the overlap function has an order of magnitude of 10^–15^, which is comparable to other donor–acceptor systems with QDs.

**Figure 3:**
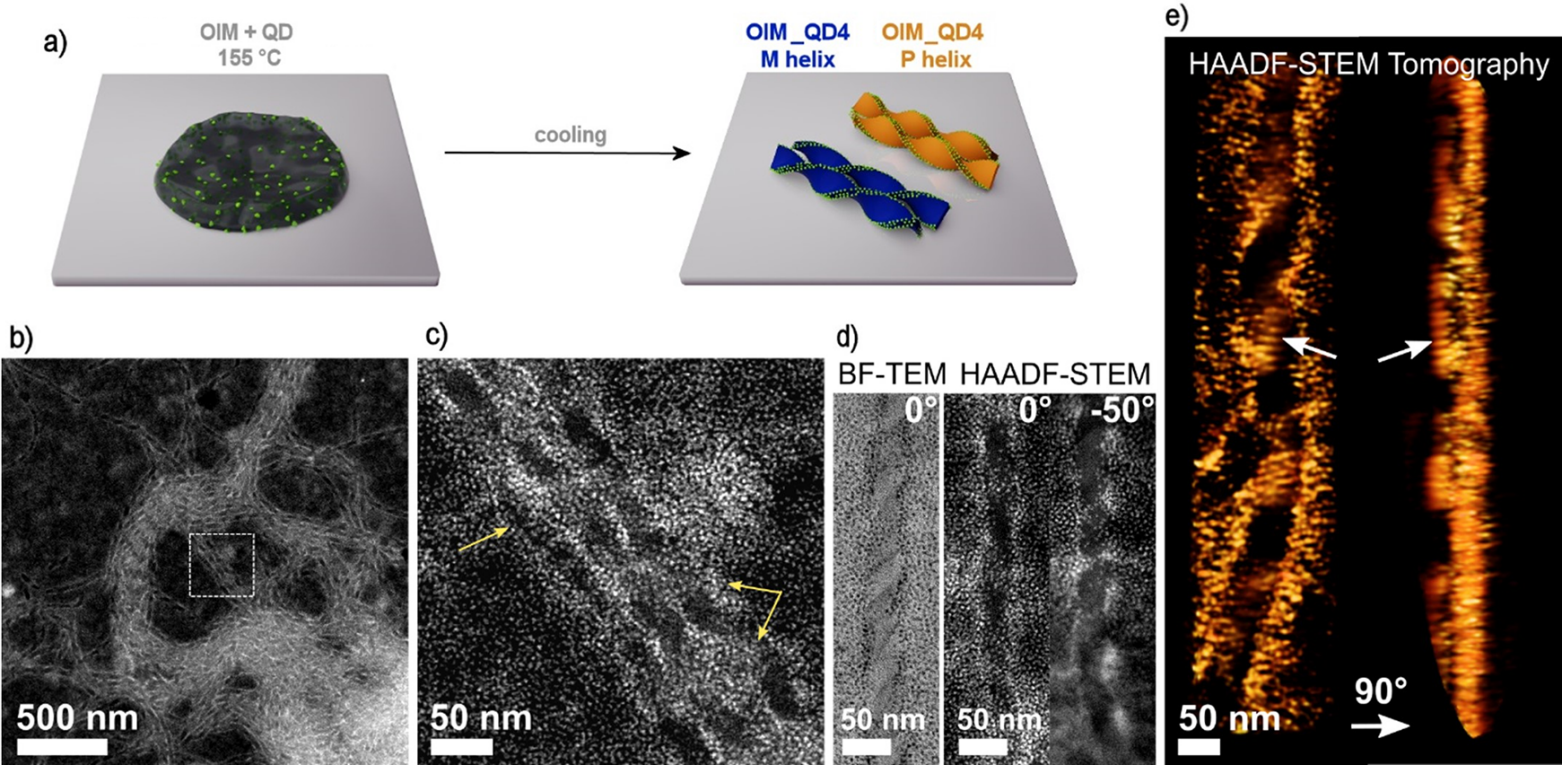
Structural analysis of OIM films doped with InP/ZnS quantum dots (OIM_QD4 composite films). (a) OIM function as a template inducing helical (chiral) arrangement of QDs by a selective decoration of helical nanofilament edges. (b) HAADF-STEM helical nanofilaments decorated with QDs. (c) HAADF-STEM image shows a magnification of an area indicated in (b) a white dashed square. A few examples of helically arranged assemblies of QDs are indicated by yellow arrows. (d) BF-TEM (left) and HAADF-STEM images (right) of individual helical nanofilaments at different tilting angles. (e) HAADF-STEM tomography reconstruction of a helical nanofilament decorated with QDs. Images at two orthogonal angles are shown. Arrows indicate the same area of the helix which features a 1D periodic, linear arrangement of QDs. One turn is highlighted as an example by white arrows in both images.

Next, we drop-casted and heat-annealed films comprising OIM and 4.5 or 8.5 wt % QDs, named OIM_QD2 and OIM_QD4 and performed a detailed structural analysis of these samples (Figure 3a, Table 1). To verify if QDs are well dispersed within the OIM matrix, small-angle X-ray scattering measurements were performed (Figure S27). An X-ray diffraction peak centered at ∼6.5 nm was detected, which can be ascribed to the formation of molecular layers by OIM (these layers, upon frustration, form helical nanofilaments), suggesting that QD presence is not preventing OIM assembly. Moreover, a broad scattering (form factor) was detected, which is not present in the diffractogram collected for undoped OIM film, suggesting that nanocrystals are well dispersed within the sample volume.

**Table 1:** Amount of QDs in Organic/Inorganic Composites^a^

sample	OIM_QD2	OIM_QD4	OIM_R/S__QD1	**OIM_R/S__QD2**	OIM_R/S__QD3	**OIM_R/S__QD4**	OIM_R/S__QD5
wt % QDs	4.5	8.5	2.3	**4.5**	6.5	**8.5**	10.5

a Samples marked in bold were used for a detailed examination of chiroptical properties.

To further verify the structure of OIM_QD2 and OIM_QD4 samples, we used 2D bright-field TEM and HAADF-STEM images to confirm that QDs were distributed within the volume of the material (Figure 3b). BF-TEM is a more adequate model to visualize the organic matrix in contrast with the QDs, whereas HAADF-STEM highlights the InP/ZnS QDs due to their high average Z atomic number. They predominantly formed twisted, 1D aggregates with dimensions characteristic of helical nanofilaments (pitch ∼220 nm, width ∼41 nm) indicating that indeed QDs are decorating helical nanofilaments (Figure S22). We estimate that ∼80% of QDs is deposited on helical nanofilaments. Given that our estimates are based on thin areas imaged with BF-TEM, comprising a limited amount of OIM, we expect that this is a lower boundary for bulk samples.

To confirm the 3D structure of assemblies, we acquired a tomography series in the HAADF-STEM mode, which has been a previously used technique to investigate the 3D morphology of helical structures at the nanoscale^73,74^ (Figure S23). The 3D reconstruction revealed ensembles of QDs adopting a helical geometry (Figure 3c). These measurements additionally revealed the hierarchical structure of assemblies, with periodicities ranging from hundreds of nanometers (pitch of the helix) to single nanometers (distance between rows of QDs, as indicated with arrows in Figure 3c).

Overall, structural analysis of OIM_QD2 and OIM_QD4 films using X-ray and electron microscopy studies proved chemical compatibility between the composite constituents, leading to the distribution of QDs within the composite, as well as the formation of chiral assemblies of QDs guided by organic helical nanofilaments.

The above-discussed analyses allowed us to conclude that OIM_R/S__QD materials represent structures of closely packed luminophores adopting a uniform, chiral morphology. We thus expected that doping QDs to OIM_R/S_ could modify the chiroptical properties of OIM_R/S_ due to the spectral matching of donor–acceptor type; specifically, that QDs could serve as energy acceptors.

**Figure 4:**
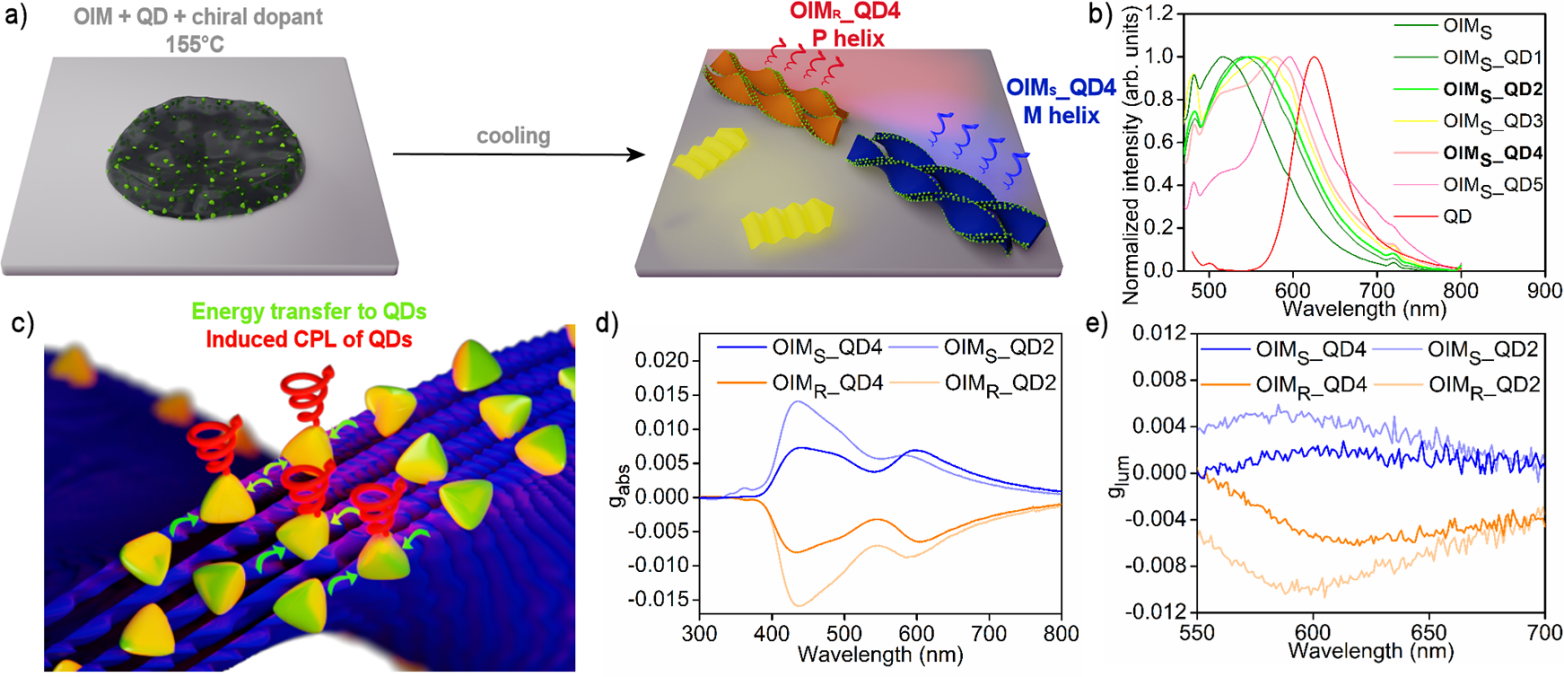
Chiroptical properties of OIM films doped with InP/ZnS quantum dots and chiral dopants (OIM_R/S__QD composite films). (a) Scheme showing the assembly of OIM_R/S_ doped with 5 wt % chiral dopants and QDs. After heat annealing (heating to 155 °C - a melted state, and cooling to 30 °C) helically arranged QDs exhibiting circularly polarized luminescence (CPL) are shown. Handedness of the helix is determined by the chiral dopant. Either P or M helices were obtained in a given sample by doping a chiral dopant of the opposite handedness. (b) Normalized PL spectra of OIM_S__QD composite films with increasing QDs wt % content from OIM_S__QD1 to OIM_S__QD5. (c) Scheme showing energy transfer (marked in green) from organic material (OIM_R/S_, blue) to InP/ZnS QDs (yellow), as well as induced CPL of QDs (marked in red). (d, e) Circular dichroism and circularly polarized luminescence dissymmetry factors, *g*_abs_, and *g*_lum_, for two selected contents of QDs in samples (OIM_R/S__QD2 and OIM_R/S__QD4; the ellipticity spectra are provided in Figure S32).

To experimentally examine these predictions, we prepared and characterized two series of multicomponent films varying (1) the amount of QDs (2.3–10.5 wt %, named OIM_S__QD1-OIM_S__QD5; details are given in Table 1), as well as (2) the handedness of added chiral dopant. Samples were drop-casted onto a glass substrate and thermally annealed, forming ∼1 μm films. We discuss in detail the analysis of samples with the S dopant, as both enantiomers give similar results. PL spectra of as-prepared composite films revealed emission bands with maxima placed in between those characteristic for pure OIM_S_ and QDs samples (Figure 4b). It is worth starting the analysis with samples having the highest QD content: OIM_S_ _QD3, OIM_S_ _QD4, and OIM_S_ _QD5. The emission spectra of these samples could be deconvoluted into two emission bands. Based on the position of maxima, the first band, λ_max_ ∼ 513 nm, could be ascribed to the emission of OIM_S_ while the second, λ_max_ ∼ 562–596 nm, to the emission of QDs. This assignment of bands is further strengthened by the growing ratio of red to green emission with the growing content of QDs. Following this interpretation, the seemingly single emission bands detected for OIM_S__QD1 and OIM_S__QD2 are most probably a sum of OIM_S_ and QD overlapping bands. Overall, the PL of OIM_S__QD materials suggested an energy transfer from OIM to QDs. To verify if the observed phenomenon was a resonant process, we measured the lifetime of OIM_S_ emission for the composites. We observed that decorating helical nanofilaments with QDs results in a shortening of OIM_S_ PL lifetime (Figures S28 and S29) which supports the resonant character of the phenomena.^13^

In the final step, we examined the chiroptical properties of multicomponent films, which we expected to originate from the above-discussed chiral, helical morphology, and OIM_S_ to QD energy transfer (Figure 4c). For this purpose, we focused on two samples that differ twice in QD wt % and exhibited the ET, doped with either R or S dopant: OIM_R/S_ _QD2 and OIM_R/S_ _QD4.

CD spectroscopy of ∼1 μm thick, heat-annealed multicomponent films revealed that irrespective of the QD amount and dopant used, the collected spectra were almost identical mirror images, with the main band placed at ∼435–438 nm (Figure 4d, Table S1). This band was previously detected in purely organic, OIM_S/R_ films and ascribed to the formation of helical nanofilaments; given the TEM analysis of multicomponent systems which revealed helical nanofilaments, the presence of this band was not surprising. The maximal absolute value of the dissymmetry factor of that band decreased with the increasing content of QDs, that is, in the order OIM_S_, OIM_S__QD2, and OIM_S__QD4 (|*g*_abs_| ∼ 0.016, 0.014, 0.007, respectively), which might reflect the inclusion character of QDs translating to a decreased order within the material. Importantly, beyond the organic-derived CD band, CD spectra of samples comprising QDs revealed additional bands in the 530–650 nm range, that is, in the absorption range of QDs. These bands appear to exhibit Cotton characteristics, although not changing signs due to an overlay onto a stronger OIM band. Notably, the measured *g*_abs_ was independent of the film thickness as confirmed by measurements of samples with 1, 4, and 10 μm thickness (Figure S30).^6^

CPL spectroscopy revealed that all tested multicomponent films were CPL active (Figure 4e and Figure S2). For a given content of QDs, but different chiral dopants, collected spectra were mirror images. With the increasing amount of QDs, two general tendencies were observed. One was shifting of the band maxima toward longer wavelengths: ∼590 vs ∼620 nm for the R dopant and ∼588 nm vs ∼617 nm in the case of the S dopant, which is in line with UV–vis, PL, and CD measurements, reflecting the higher coupling between QDs. With growing QDs content, the absolute dissymmetry factors decrease, |*g*_lum_| ∼ 0.005 vs 0.002 for the S dopant and ∼0.01 vs ∼0.006 in the case of the R dopant. The detected decrease may be the result of the growing fraction of QDs not deposited onto helical nanofilaments (Figures S24 and S25). Overall, up to 90 nm redshift of CPL bands upon doping the film with QDs goes in line with TEM, CD, PL, and UV–vis results, attesting the achievement of energy transfer from chiral OIM_S_ to achiral QDs and induced chiroptical properties of QDs. Notably, the measured *g*_abs_ value is independent of the film thickness as confirmed by measurements of samples with thicknesses of ∼4 and ∼10 μm (Figure S30).^6^

**Figure 5:**
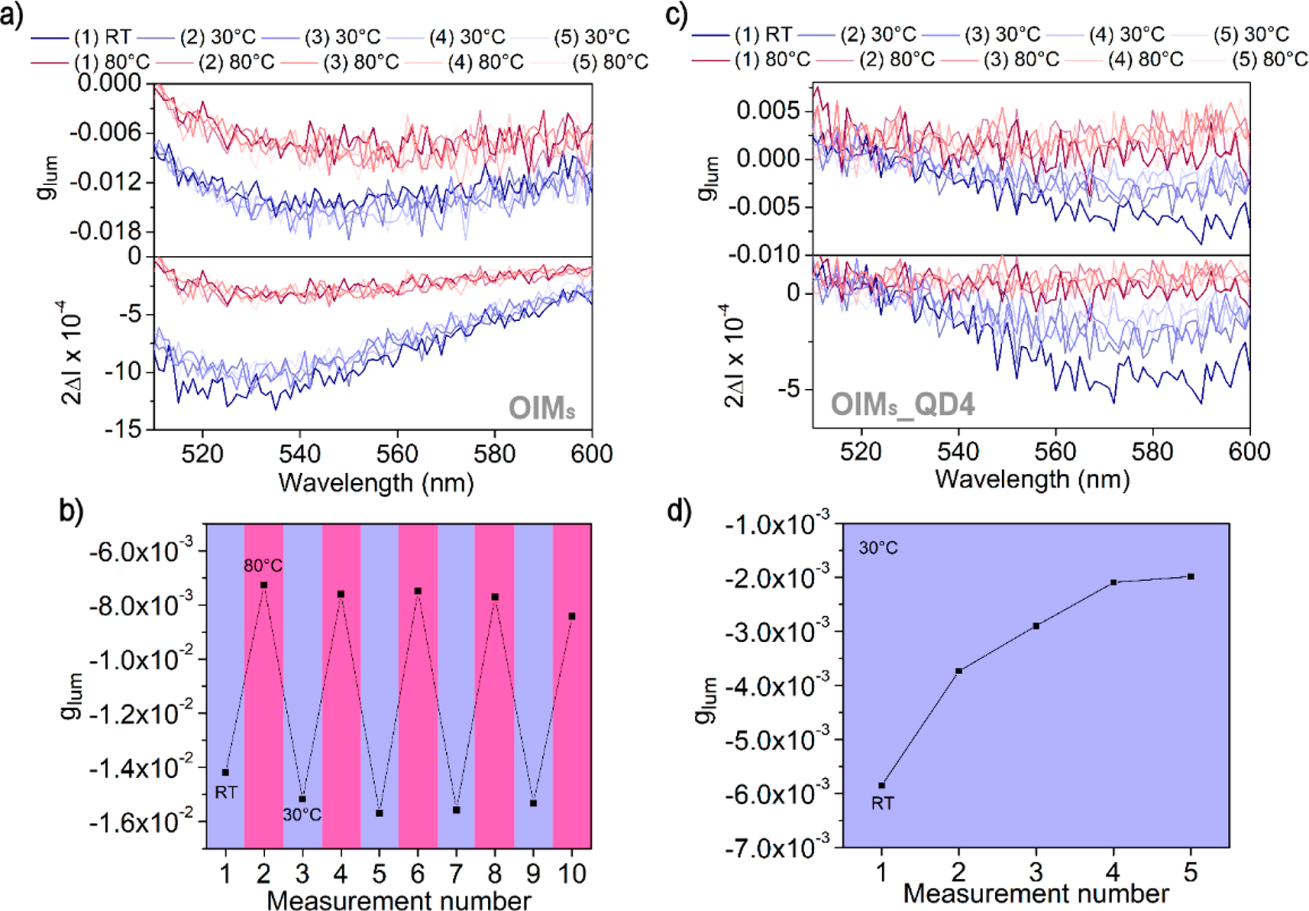
Temperature dependence of CPL properties of purely organic and composite films. (a) Reversible variation of OIM_S_ CPL spectra collected in consecutive heating/cooling cycles (30–80 °C) given as *g*_lum_ and 2Δ*I*. The ellipticity spectra are provided in Figure S33. (b) Reversible variation of OIM_S_ CPL measured in heating/cooling cycles (30–80 °C). The values are averaged across the 550–570 nm spectral range. (c) Reversible variation of OIM_S_ CPL spectra collected in consecutive heating/cooling cycles (30–80 °C) given as *g*_lum_ and 2Δ*I*. (d) *g*_lum_ value of OIM_S__QD4 in 30 °C in consecutive heating/cooling cycles (30–80 °C). The values provided were recorded at 30 °C. Values recorded at 80 °C are not reported due to a thermal quench of the emission, resulting in almost nullified 2Δ*I*; thus *g*_lum_ is assumed as 0.

In the final stage of research, we decided to examine whether helical nanofilaments decorated with QDs exhibit switchable CPL properties. For this purpose, composite films deposited onto glass substrates were mounted to a heating stage within the CPL spectrometer. CPL spectra of purely organic, OIM_S_, and composite, OIM_S__QD4, films were recorded directly after preparation and in five consecutive heat/cool cycles in which temperature was varied between 30 and 80 °C (Figure 5). In the case of the purely organic, OIM_S_ sample, a reversible change in the CPL signal intensity (from ∼0.08 for the first measurement performed at room temperature to ∼0.04 at 80 °C and ∼0.06 at 30 °C) and dissymmetry factor (∼1.5 × 10^–2^ at 30 °C and ∼7 × 10^–3^ at 80 °C) was recorded. In the case of composite, OIM_s__QD4 sample, increasing the temperature to 80 °C results in a decrease of the overall emission and therefore of the CPL properties to almost zero. The *g*_lum_ values at 80 °C are solely derived from instrumental noise and should be considered as 0. However, the sample recovers the CPL properties when cooled to 30 °C, preserving the *g*_lum_ values in consecutive heat–cool cycles. We could also observe that the *g*_lum_ of OIM_S__QD4 reaches a stable value at 30 °C over the heat–cool cycles (Figure 5d). We also examined how the CPL intensity changes with the gradual change of temperature (Figure S34). Due to the broad spectra of fluorescence, we determined the intensity and *g*_lum_ in two manners: pointwise at 560 nm and averaging intensity in the range 550–570 nm. Both methods give comparable results; the intensity and dissymmetry factor change linearly. This indicates that although the origin of the CPL phenomena in discussed systems is the aggregation of OIM molecules, it can be successfully thermally quenched.

## Conclusion

Loading InP/ZnS quantum dots into an emissive liquid crystal was established as a simple way to realize CPL active films. We have confirmed that liquid crystal forming morphologically chiral helical nanofilaments exhibits AIE-CPL. This liquid crystal can effectively serve as a helical matrix guiding assembly of doped QDs, inducing chiroptical properties of QDs. Moreover, energy transfer from organic to inorganic constituents enabled a convenient way of CPL spectral tuning via varying the amount of added QDs. Finally, we disclose reversible thermal quenching of CPL coming from liquid crystal-quantum dots composites. The presented approach affords deep insight into the chirality induction and energy transfer in nanocomposite systems and should be widely applicable for designing functional organic/inorganic CPL-active materials.

## Experimental Section

### Chemicals

All chemicals were used before and used as purchased, without any further purification: InCl_3_ (Sigma-Aldrich, 99.999% trace metal basis), ZnCl_2_ (Sigma-Aldrich, 99.999% trace metal basis), oleylamine (OAm, Acros, 80–90% grade), tris(diethylamino)phosphine ((DEA)_3_P, Sigma-Aldrich, 97%), sulfur powder (Sigma-Aldrich, ≥99.0%), trioctylphosphine (TOP, Sigma-Aldrich, 97%), zinc stearate (ZnSt_2_ Sigma-Aldrich, technical grade), 1-octadecence (ODE, Alfa Aesar, 90%), R5011 and S5011 (Henan Tianfu Chemical, 99%). All reagents and solvents used in L, OIM, and P-8-OPIMB synthesis were obtained from Sigma-Aldrich.

### Synthesis of InP/ZnS QDs and Functionalization

InP/ZnS QDs synthesis was performed according to the previous report.^66^ 100 mg (0.45 mmol) of InCl_3_, 300 mg (2.2 mmol) of ZnCl_2_, and 5 mL of OAm were mixed in a three-necked flask and heated to 120 °C for 1 h in vacuum conditions. Then nitrogen flow was opened and the temperature increased to 180 °C. 0.45 mL of (DEA)_3_P was swiftly injected. After 20 min 1 mL of sulfur stock solution (360 mg of sulfur powder in 5 mL of TOP, sonicated) was added dropwise. After 40 min the temperature was increased to 200 °C; after the next 60 min zinc stock solution (1 g of zinc stearate suspended in 4 mL of ODE) was added dropwise, and the temperature was increased to 220 °C. After 30 min 0.7 mL of sulfur stock solution was injected, and the temperature was increased to 240 °C. After 30 min 2 mL of zinc stock solution (0.5 g of zinc stearate suspended in 2 mL of ODE) was added and the temperature increased to 260 °C. The reaction was stopped after 30 min by removing a heating mantle and cooling the reaction vessel at room temperature. 20 mL of toluene (used as received) was added to the reaction mixture and centrifuged (7000 rpm for 5 min) to remove an excess of organic compounds. Then, supernatant was discarded and QDs aggregate was dispersed in toluene and a small portion of acetone (antisolvent for QDs) was added. In the presence of antisolvent, the largest QDs become unstable in the dispersion and can be separated by centrifugation. The dispersion was centrifuged (7000 rpm for 5 min), yielding a fraction of size-focused QDs and redispersed in toluene. The procedure for size fractioning was repeated by adding additional portions of acetone to the supernatant and consecutive centrifugations. A fraction of 3.5 ± 0.4 nm QDs was selected for further functionalization.

In the next step, QDs were modified with a monolayer of dodecanethiol/L ligands using a two-step ligand exchange reaction described in the previous report.^75^ A 3-fold molar excess of dodecanethiol was added to InP/ZnS capped with OAm dispersion in toluene and left overnight in a glass vial, ensuring magnetic stirring. After this time, to remove the unbound ligands, the QDs were precipitated several times with acetone and dispersed in toluene. For partial modifying of DDT-coated QDs’ surface with L ligand (chemical structure in Supporting Information), a ligand exchange reaction was conducted. The purified QDs (0.5 mL, 10 mg mL^–1^) were mixed with a toluene solution of liquid crystal-like ligand (L, 2.0 mL, 10 mg mL^–1^) and left on stirring (400 rpm) for 1 h. The final QDs were achieved by purifying the ligand exchange mixture, followed by adding acetone as antisolvent, centrifugation (4 000 rpm, 5 min), and dispersion in toluene.

### Helical Nanofilaments Preparation

Samples were prepared on glass coverslips, previously washed with soap, water, and rinsed with acetone. Bulk solutions of OIM and chiral dopants were prepared. For OIM_R/S_ samples 270 μL of OIM solution (3 mg/mL, THF) was added to 45 μL of dopant (1 mg/mL, THF) and subjected to a flow of air with a temperature of 80 °C, to reach the final volume of ∼20 μL. The mixture was drop-casted on a glass substrate and heat-annealed using a temperature control stage. The heat annealing procedure included two heat–cool cycles. In the first stage, samples were heated to 155 °C and cooled to 30 °C, then heated to 80 °C and cooled to 30 °C. The temperature changes were conducted at a rate of 3 °C/min.

To prepare helical nanofilaments decorated with QDs, QDs dispersion is added to the solution of OIM and dopant. In detail, 270 μL of OIM solution (3 mg/mL, THF) was added to 45 μL of dopant (1 mg/mL, THF), and the QDs dispersion (1 mg/mL, toluene) was added in various wt % ratios to the organic matrix (1–12%, Table 1). The prepared mixtures were also concentrated and heat-annealed as mentioned above.

### Structural Characteristics

Thermal annealing was conducted using a temperature control stage Linkam TP 93 with 0.1 K resolution. BF TEM images were taken using a transmission electron microscope JEM-1011 (JEOL) equipped with a model EDS INCA analyzer (Oxford, U.K.), in the Electron Microscopy Platform, Massakowski Medical Research Centre, Polish Academy of Science, Warsaw. Scanning electron microscope analysis was performed using a Zeiss LEO 435VP instrument with a tungsten cathode available at the Faculty of Chemistry, University of Warsaw.

### Transmission Electron Microscopy

HAADF-STEM imaging and tomography were performed using an aberration-corrected Thermo Fisher Scientific Titan Cubed electron microscope, operated at 300 kV (Electron Microscopy for Materials Research, Antwerp). HAADF-STEM tomography tilt series were acquired using a Fischione model 2020 single-tilt tomography holder. The heating procedure on the QDs was performed using a Wildfire DENS Solutions heating holder, and the sample was heated to 180 °C. A DENSsolutions Wildfire heating sample holder optimized for electron tomography was used. Tomographic series were acquired within a ±70° tilt range and a tilt increment of 3°.

To eliminate different image distortions, we applied a convolutional neural network (CNN). Undistorted images were aligned with respect to each other by using a phase correlation, which was also used to determine the shift and the angle of the rotation axis. 3D reconstruction shown in Figure 3 was performed using a simultaneous iterative reconstruction technique (SIRT) algorithm, as implemented in Astra Toolbox.^76^ 3D reconstruction in Figure S23 was performed by using an approach consisting of iterating between several SIRT cycles and application of constraints in the real and Fourier space to obtain a high-quality 3D reconstruction with a diminished missing wedge.^74,77^

### Optical Characteristics

Photoluminescence was measured with a modified Fluorolog 3-2-IHR320-TCSPC Horiba-Jobin Yvon fluorimeter equipped with front face detection mode (Faculty of Chemistry, University of Warsaw). PL data were collected and processed with FluorEssence software. CD measurements were performed using a Chirascan circular dichroism spectrometer, available at the University of Warsaw. Spectroscopy studies in the UV–vis range were performed using a GENESYS 50 UV–vis spectrophotometer, available at the Faculty of Chemistry, University of Warsaw.

CPL measurements were performed using a JASCO CPL-300 spectrophotometer, equipped with a 150 W Xe lamp as a light source in 180° geometry. The thin film samples on glass substrates were mounted so that the sample plane was normal to the excitation beam. CPL spectra of OIM and OIM_R/S__QD composite films were recorded with an excitation and emission bandwidth of 25 nm and an excitation wavelength of 440 nm. Each sample was measured in four rotated positions (0°, 90°, 180°, and 270° around the axis defined by the excitation beam with irradiation directly onto the composite film) and flipped (excitation beam hitting the glass first and then the composite film). Each orientation was averaged over 40 spectra (1 nm steps, 0.5 s digital integration time), and finally, the four rotations were averaged to exclude orientation-dependent artifacts. Temperature-dependent CPL spectra were averaged over 20 spectra and measured in a single orientation. The heating and cooling cycles and temperature control were performed upon inserting a heating plate with an aperture allowing probing of the film (temperature control stage Linkam TP 93 with 0.1 K resolution) in the sample chamber of the CPL instrument, keeping the same geometry for the measurements.

## Supporting Information Available

Supplementary notes on rationale for the choice of organic compounds used in the study, synthesis and structural characteristics of organic compounds, phase sequences of materials and composites used in the study, physicochemical characteristics of OIM compound forming helical nanofilaments (1,3-phenylenebis[4-(4-oleyloxyphenyliminonetyl)benzoate]), synthetic procedures of organic compounds; supplementary figures showing NMR and MS spectra, POM, DSC and X-ray analysis of the organic compounds, additional CPL and CD spectra of OIM_R/S_ and OIM_R/S__QD4, additional TEM images of helical composites, 3D reconstruction of the composite helical nanofilaments, PL spectra of OIM in solution and thin films, TGA analysis of InP/ZnS QDs, and SAXRD patterns of helical nanofilaments, and POM images and DSC curves of organic compounds and composites (PDF)
