# Supplementary material for: Tunable Circularly Polarized Luminescence via Chirality Induction and Energy Transfer from Organic Films to Semiconductor Nanocrystals
Source: ACS Nano. 2022 Nov 7;16(11):18472–82. doi: 10.1021/acsnano.2c06623 (PMC9706675; doi:10.1021/acsnano.2c06623)
Supplement: Supplementary file 1 — nn2c06623_si_001.pdf [file nn2c06623_si_001.pdf]

# Tuneable circularly polarized luminescence via energy transfer and chirality induction from organic films to semiconductor nanocrystals

*Sylwia Parzyszek†, Jacopo Tessarolo‡, Adrián Pedrazo-Tardajos§∥, Ana M. Ortuño‡, Maciej Bagiński†, Sara Bals§∥, Guido H. Clever‡, Wiktor Lewandowski†**

† Faculty of Chemistry, University of Warsaw, 1 Pasteur St., 02-093 Warsaw, Poland, Email: wlewandowski@chem.uw.edu.pl (W.L.)

‡ Faculty of Chemistry and Chemical Biology, TU Dortmund University, Otto-Hahn Straße 6, 44227 Dortmund, Germany

§ Electron Microscopy for Materials Research, University of Antwerp, Groenenborgerlaan 171, 2020 Antwerp, Belgium

∥ NANOlab Center of Excellence, University of Antwerp, 2020 Antwerp, Belgium

Supplementary note 1: Rationale for the choice of organic compounds used in the study .......... S3

Supplementary note 2: Synthesis and structural characteristics of organic compounds .............. S5

Supplementary note 3: Phase sequences of materials and composites used in the study ........... S10

Supplementary note 4: Physicochemical characteristics of OIM compound forming helical nanofilaments (1,3-phenylenebis[4-(4-oleyloxy-phenyliminonetyl)benzoate]) ......................... S15

Bibliography ........................................................................................................... S30

## Supplementary note 1: Rationale for the choice of organic compounds used in the study

There are several reasons for choosing the particular organic structures used in the study. Below we discuss this topic, analyzing the overall structure of compounds forming helical nanofilament phase, as well as giving reasons for choosing OIM, R5011 and S5011 compounds.

Helical nanofilament phase is formed by bent-core mesogenic molecules assembling into layers. Due to the incompatibility of packing of the aromatic, mesogenic arms of the molecules in layers, a local preference for saddle splay curvature of these molecular layers arises with a radius comparable to only a few layers, leading to the formation of helical nanofilaments. Many examples of bent-core molecules assembling into the helical nanofilament phase have been described in the literature. A common structural motif is the presence of two main structural elements: a central unit which ensures the bent shape of the molecule (alkyl chain,[1–3] m-substituted benzene,[4–6] 3,2'-substituted binaphthyl[5]), as well as two mesogenic units attached to the central unit. These mesogenic units are composed of aromatic rings linked by bonds that usually exhibit a limited degree of rotation, but this is not a prerequisite. The mesogenic units are equipped with a terminal alkyl chain.

<u>The reasons for using OIM compound</u> as the matrix material in comparison to other molecules forming the helical nanofilament phase:

(1) a relatively simple and straightforward synthesis, relying, e.g., on the ease of obtaining imine bonds,

(2) the high tendency of molecules of this class (a series called P-n-OPIMB)[7] to form helical nanofilaments; particularly, this tendency is higher than that of a dimer compound with which we worked before;[8] namely, OIM does not exhibit a preference to form non-twisted layered structures,

(3) we modified the basic design of P-n-OPIMB bearing saturated terminal chains by including an oleyl terminal chain; this modification lowers isotropization temperature by ~40 °C in comparison to other members of P-n-OPIMB family equipped with saturated terminal chains; this is a crucial modification since melting the matrix to the isotropic phase is an obligatory step for introducing quantum dots onto the surface of the helical fibers; the high

melting point of the organic matrix material would be a significant disadvantage, as it could lead to the reshaping/agglomeration of doped nanoparticles.

The reasons for using R5011 and S5011 compounds as chiral dopants:

(1) achieving preferential twisting (preferential symmetry breaking) in helical nanofilament systems is a non-trivial problem; a few approaches have been studied towards this goal, which are dependent on using: compounds bearing an asymmetric carbon atom,[9,10] chiral dopants,[11,12] chiral surfaces,[13,14] circularly polarized light,[15,16] or twisted nematic cells[17]. We decided to use chiral dopants for symmetry breaking, as this methodology is substrate independent, and was already proven successful in the case of composites of gold nanoparticle doped to helical nanofilaments.

(2) usually, chiral dopants having an asymmetric carbon atom were used for symmetry breaking, however, using dopants showing axial chirality is common for LC and polymer systems;[18,19] we envisage the chiral dopant molecules interact with OIM molecules in the crystallization points, inducing (small) energetic inequivalence for the right and left-handed filaments; handedness adopted at the crystallization point is then propagated through chirality synchronization phenomena,

(3) the R5011 and S5011 dopants turned out to more effectively drive the symmetry breaking than rod-like dopants with an asymmetric carbon atom used in our previous study,[12] allowing us to limit the wt% of the chiral dopant in the composite in comparison to the previous work;

(4) the R5011 and S5011 chiral dopants do not form ordered films (they form a glassy state on cooling from the isotropic phase), thus their addition to the OIM does not introduce birefringence, which could potentially complicate the analysis of circular dichroism measurements.

## Supplementary note 2: Synthesis and structural characteristics of organic compounds

4-[(16-sulfanylhexadecanoyl)oxy]phenoxy-4-(hexadecyloxy)benzoate (**L ligand**, Figure S1) was synthesized and used as a ligand ensuring chemical compatibility of nanoparticles with the organic matrix. This compound is analogous to the molecule previously used by us when working with plasmonic nanoparticles.[5,20] One difference is that here we use a shorter alkyl linker (the one bearing the thiol moiety) to shorten the synthetic protocol.

1,3-phenylenebis[4-(4-oleyloxy-phenyliminonetyl)benzoate] (**Oleyl-Imine-Matrix, OIM**) was synthesized and used as the matrix for NCs organization (Figure S1b). OIM structure is similar to the well-investigated family of liquid crystals efficiently forming helical nanofilaments (P-n-OPIMB)[7,12,20].

**P-8-OPIMB** (compound analogous to OIM with 8-carbon long terminal alkyl chains) was obtained according to the literature procedure.[7,12,20]

Below we present synthetic schemes, copies of spectra and their analyses for the final compounds (L and OIM). L and OIM (as well as all intermediate compounds) were obtained according to the procedure described in our previous work, with the difference that cetyl alcohol was used instead of an oleyl alcohol in the first synthetic step.[12]

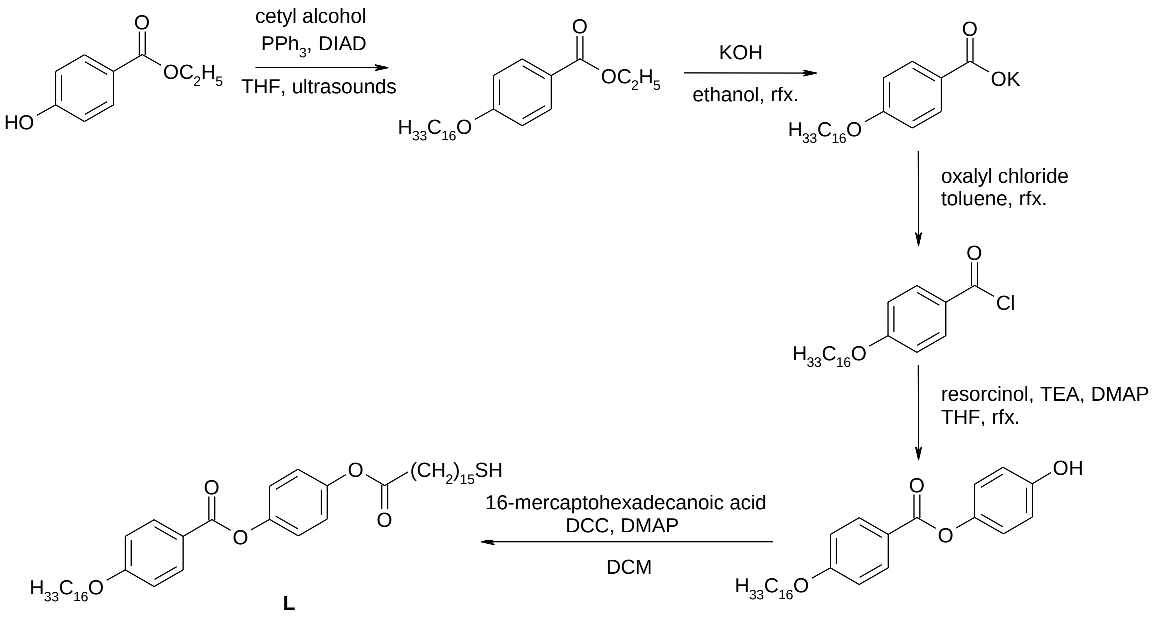


Figure S1. The chemical structure and scheme of synthesis of a liquid crystalline-like ligand L.

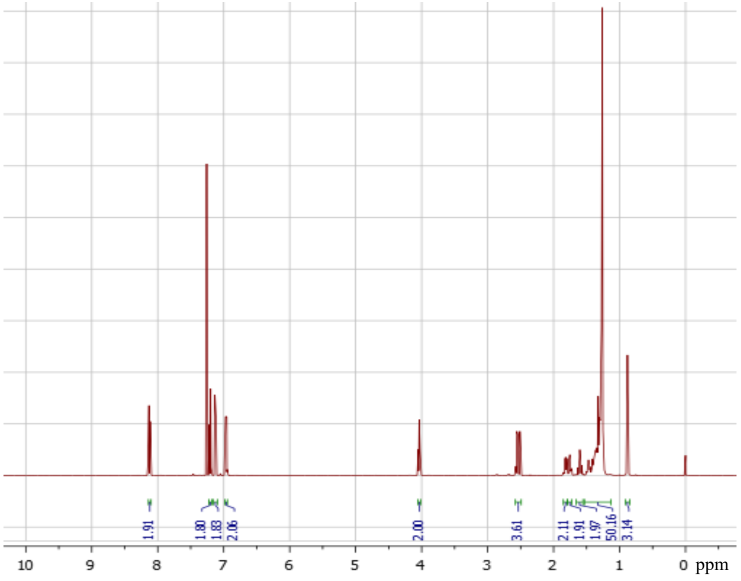


Figure S2. 1H NMR spectra of a liquid crystalline-like ligand L.

**1H NMR of ligand L** (500 MHz, CDCl3, 25 °C, TMS): $\delta$ = 8.12 (d, J= 9.0 Hz, 2H; ArH), 7.40 (t, J= 6.4 Hz, 1H; ArH), 7.11-7.08 (m, 1H; ArH), 7.03-6.99 (m, 2H; ArH), 6.98-6.94 (d, J= 9.0 Hz, 2H; ArH), 4.04 (t, J= 6.5 Hz, 2H; OCH2), 2.57-2.49 (m, 4H; CH2SH and OCOCH2), 1.85-1.77 (m, 2H), 1.76-1.70 (m, 2H), 1.52-1.45 (m, 4H), 1.42-1.20 (m, 46H), 0.88 (t, J= 7.0 Hz, 3H, CH3).

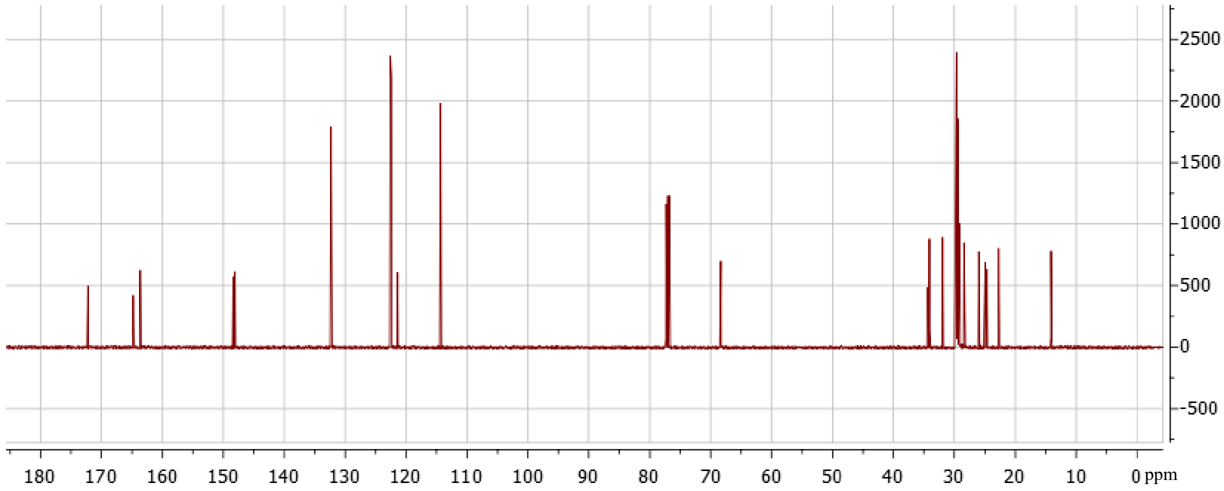


Figure S3. 13C NMR spectra of a liquid crystalline-like ligand L.

**13C NMR of a liquid crystalline-like ligand L** (500 MHz, $CDCl_3$, 25°C, TMS): δ = 171.95, 164.55, 163.64, 151.54, 151.27, 132.32, 129.65, 121.26, 119.15, 118.86, 115.74, 114.33, 68.36, 34.40, 34.08, 31.94, 29.71, 29.69, 29.67, 29.66, 29.65, 29.60, 29.57, 29.53, 29.46, 29.38, 29.27, 29.11, 29.09, 28.40, 26.00, 24.91, 24.68, 22.71, 14.14.

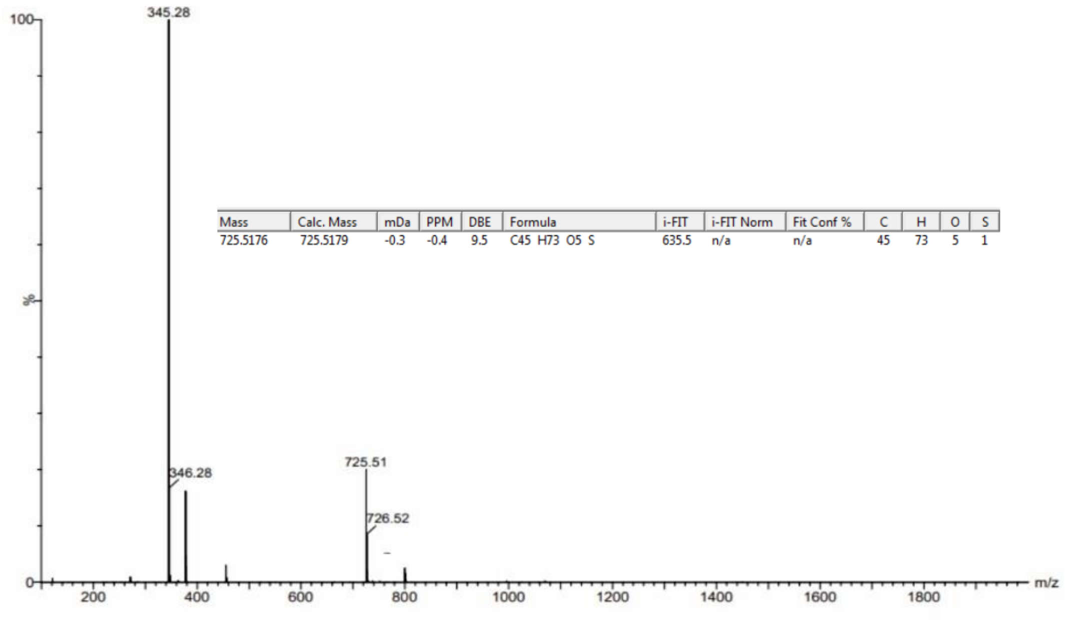


Figure S4. Mass spectrometry analysis of a liquid crystalline-like ligand L.

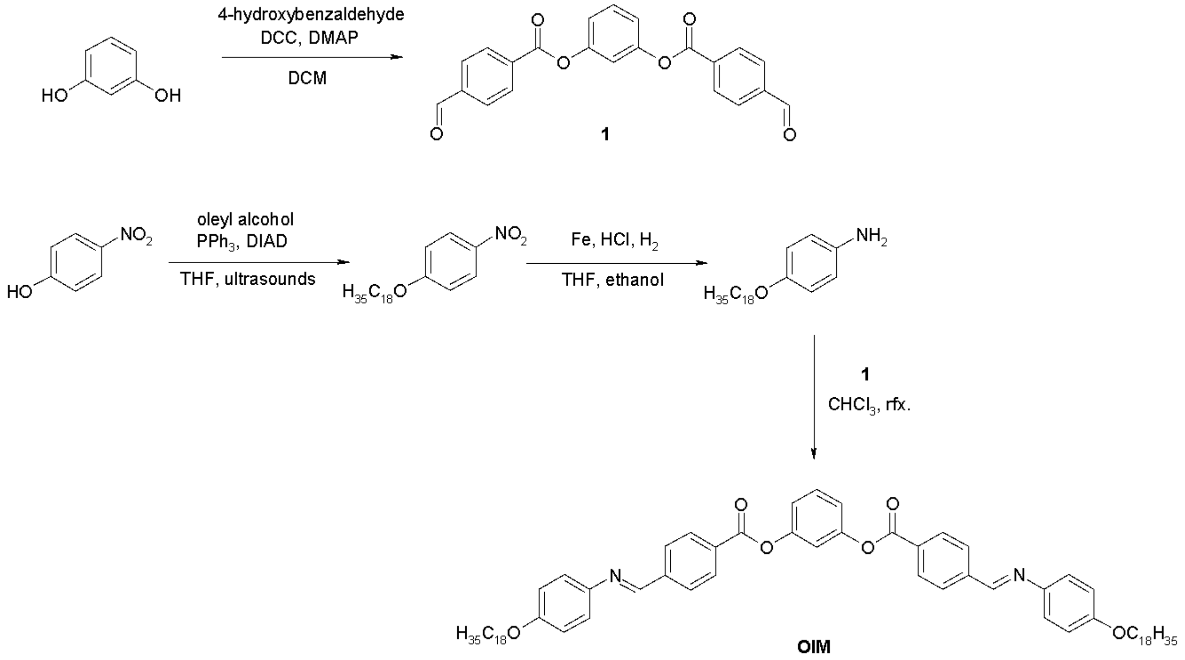


Figure S5. Chemical structure and scheme of synthesis of OIM compound forming helical nanofilaments (1,3-phenylenebis[4-(4-oleyloxy-phenyliminonetyl)benzoate]).

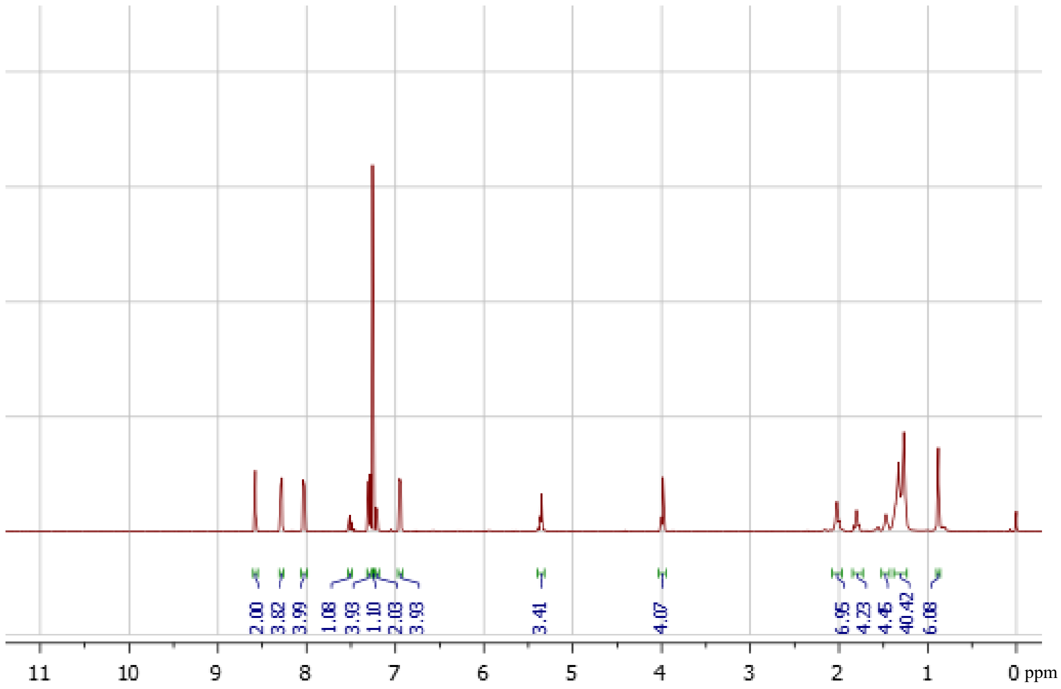


Figure S6. 1H NMR spectra of OIM compound forming helical nanofilaments (1,3-phenylenebis[4-(4-oleyloxy-phenyliminonetyl)benzoate]).

**1H NMR of OIM compound forming helical nanofilaments (1,3-phenylenebis[4-(4-oleyloxy-phenyliminonetyl)benzoate])** (500 MHz, CDCl3, 25 °C, TMS): δ = 8.60 (s, 2H; =CHAr x2), 8.31 (d, J= 8.2 Hz, 4H; ArH), 8.05 (d, J= 8.2 Hz, 4H; ArH), 7.53 (t, J= 7.8 Hz, 1H; ArH), 7.35-7.20 (m, 7H; ArH), 6.96 (d, J= 8.2 Hz, 4H; ArH), 5.38-5.30 (m, 4H, CH=CH x2), 4.01 (t, J= 7.4 Hz, 4H, OCH2 x2), 2.10-1.96 (m, 8H), 1.88-1.80 (m, 4H), 1.60-1.30 (m, 44H), 0.88 (t, J= 7.0 Hz, 6H; CH3).

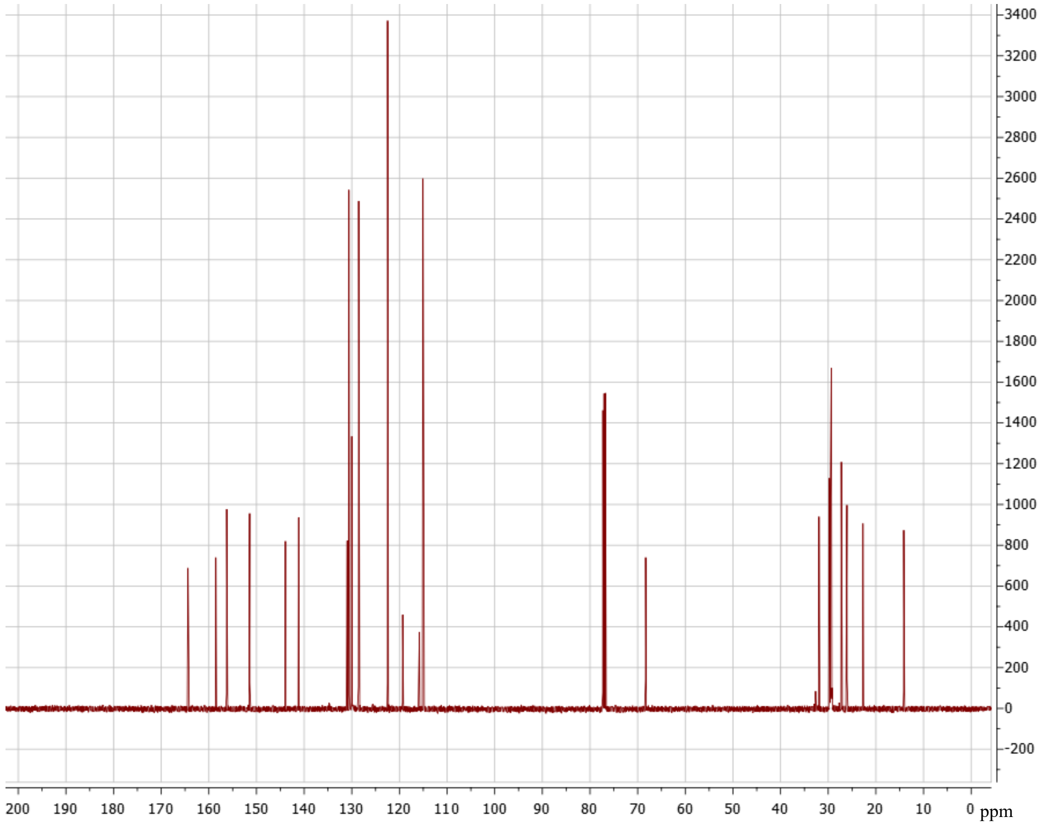


Figure S7. 13C NMR spectra of OIM compound forming helical nanofilaments (1,3-phenylenebis[4-(4-oleyloxy-phenyliminonetyl)benzoate]).

**13C NMR of OIM compound forming helical nanofilaments (1,3-phenylenebis[4-(4-oleyloxy-phenyliminonetyl)benzoate])** (500 MHz, CDCl3, 25 °C, TMS): δ = 173.99, 64.33, 63.08, 34.38, 34.08, 32.81, 32.78, 29.50, 29.49, 29.42, 29.38, 29.35, 29.32, 29.20, 29.11, 28.72, 28.63, 28.15, 25.90, 25.71, 24.99.

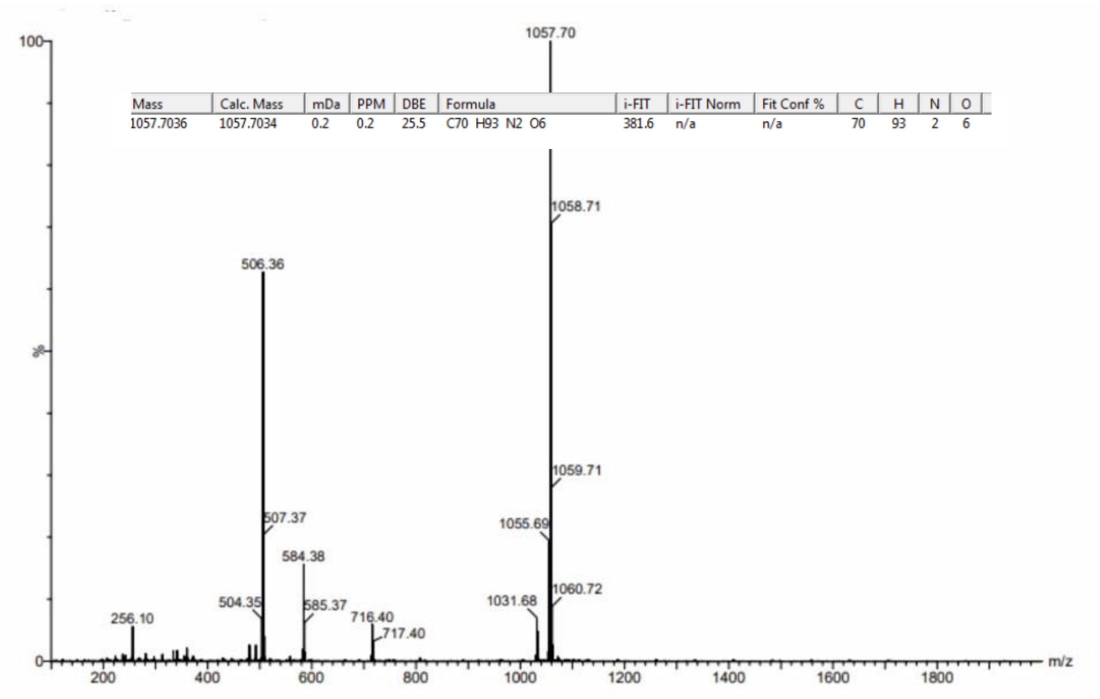


Figure S8. Mass spectrometry analysis of OIM compound forming helical nanofilaments (1,3-phenylenebis[4-(4-oleyloxy-phenyliminonetyl)benzoate]).

## Supplementary note 3: Phase sequences of materials and composites used in the study

Below we present the results of polarizing optical microscopy (POM), differential scanning calorimetry (DSC) and X-ray diffraction using General Area Detector Diffraction (GADDS) analysis of organic and composite materials obtained in this study. These analyzes allowed us to evaluate phase sequences of tested materials.

A) **L** - this compound crystallizes directly to a birefringent crystal phase; DSC measurements revealed the presence of two crystal phases (Figure S9).

   **Phase sequence**: Iso (89.9°C) Cr 2 (56.8°C) Cr1

B) **OIM** – POM measurements confirmed the formation of the previously reported B7 and B4 phases, although DSC, combined with POM and GADDS methods (at temperatures characteristic to the identified phases) enabled a precise phase assignment (Figure S10).

**Phase sequence**: Iso (138.8°C) B7 (120.3°C) B4' (110.0°C) B4

On cooling from an isotropic phase, OIM forms the liquid crystalline B7 phase. This phase can be recognized based on a characteristic texture under POM, while X-ray diffractogram reveals a narrow signal in the small-angle range, confirming the layered character of the material (layer thickness ~4.8 nm), and a wide peak in the wide-angle range, attesting liquid-like order of molecules within layers.

Further cooling of the material results in the formation of B4' phase, which exhibits optical activity characteristic to the helical nanofilament phase, while the X-ray diffractogram confirms the layered character of the phase (layer thickness ~5.6 nm); as expected for the formation of a helical nanofilament phase, X-ray diffractograms revealed broadening of the layered signal (resulting most probably from the limited thickness of helical nanofilaments) and the appearance of two wide-angle signals, indicating a more crystal-like character of the phase in comparison to the B7 phase.

Further cooling of the material results in a phase transition to the B4 (helical nanofilament) phase, which exhibits optical activity (POM texture identical to that of B4' phase), while X-ray diffractograms reveal slightly decrease layer thickness (~5.5 nm), and narrowing of the wide angle signals enabling identification of few peaks. Diffractograms collected at 30 and 80 °C are almost identical, with minute changes in the wide-angle region.

C) **chiral dopant** (R5011/S5011) – these compounds were bought and used as received; POM investigation of thin films of chiral dopants revealed they do not form liquid crystalline phases; at an ambient temperature they form non-fluid films, which appear dark under crossed polarizers, suggesting the formation of a glassy state.

D) **OIM**$_{\mathbf{S}}$ - a texture characteristic to the B7 liquid crystalline phase was revealed at 120°C, confirming LC properties of OIM doped with 5 wt% S5011 dopant; a POM texture showing the optical activity of a single domain was revealed at an ambient temperature (Figure S11).

**Phase sequence**: Iso (127.2°C) B7 (123.4°C) B4' (111.6°C) B4

E) **OIMs_QD4** – POM measurements revealed the formation of the B7 phase only for a very narrow temperature range, in agreement with DSC results (Figure S12).

**Phase sequence**: Iso (116.0°C) B4

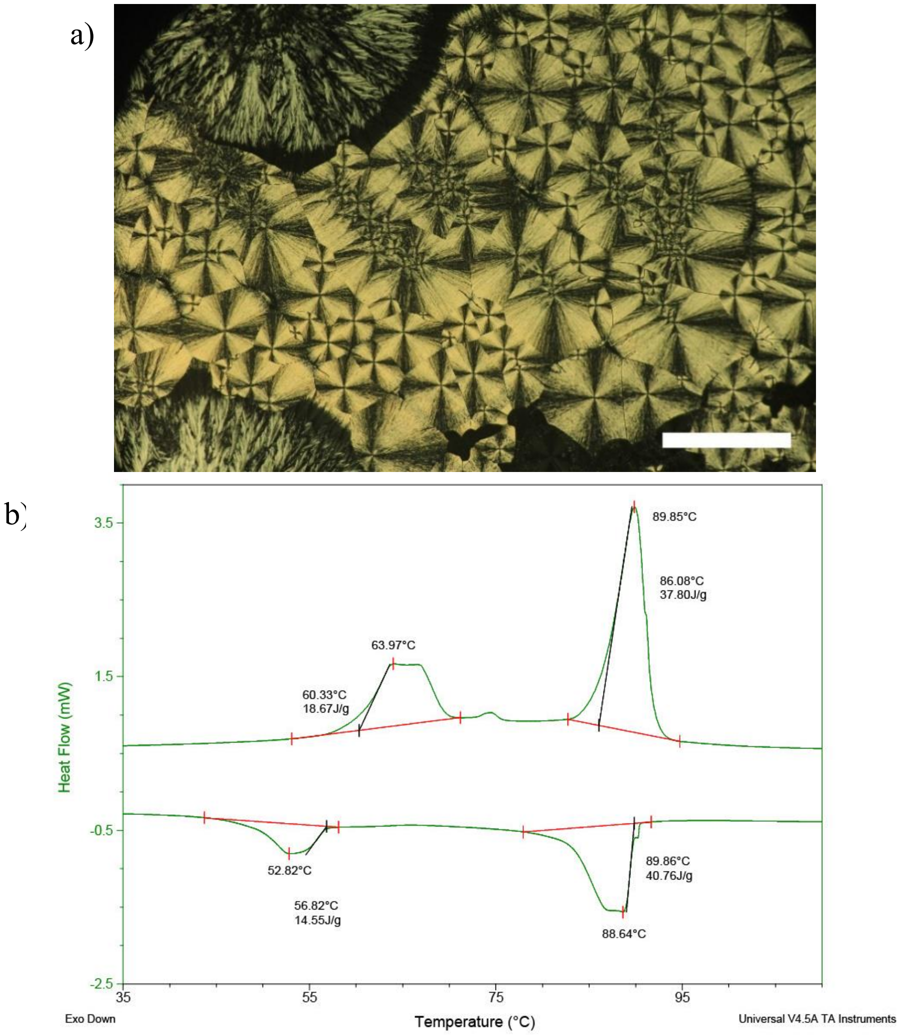


Figure S9. (a) POM image of the liquid crystal-like ligand L at an ambient temperature after thermal annealing. The scale bar corresponds to 200 μm. (b) Differential Scanning Calorimetry of the the liquid crystal-like ligand L.

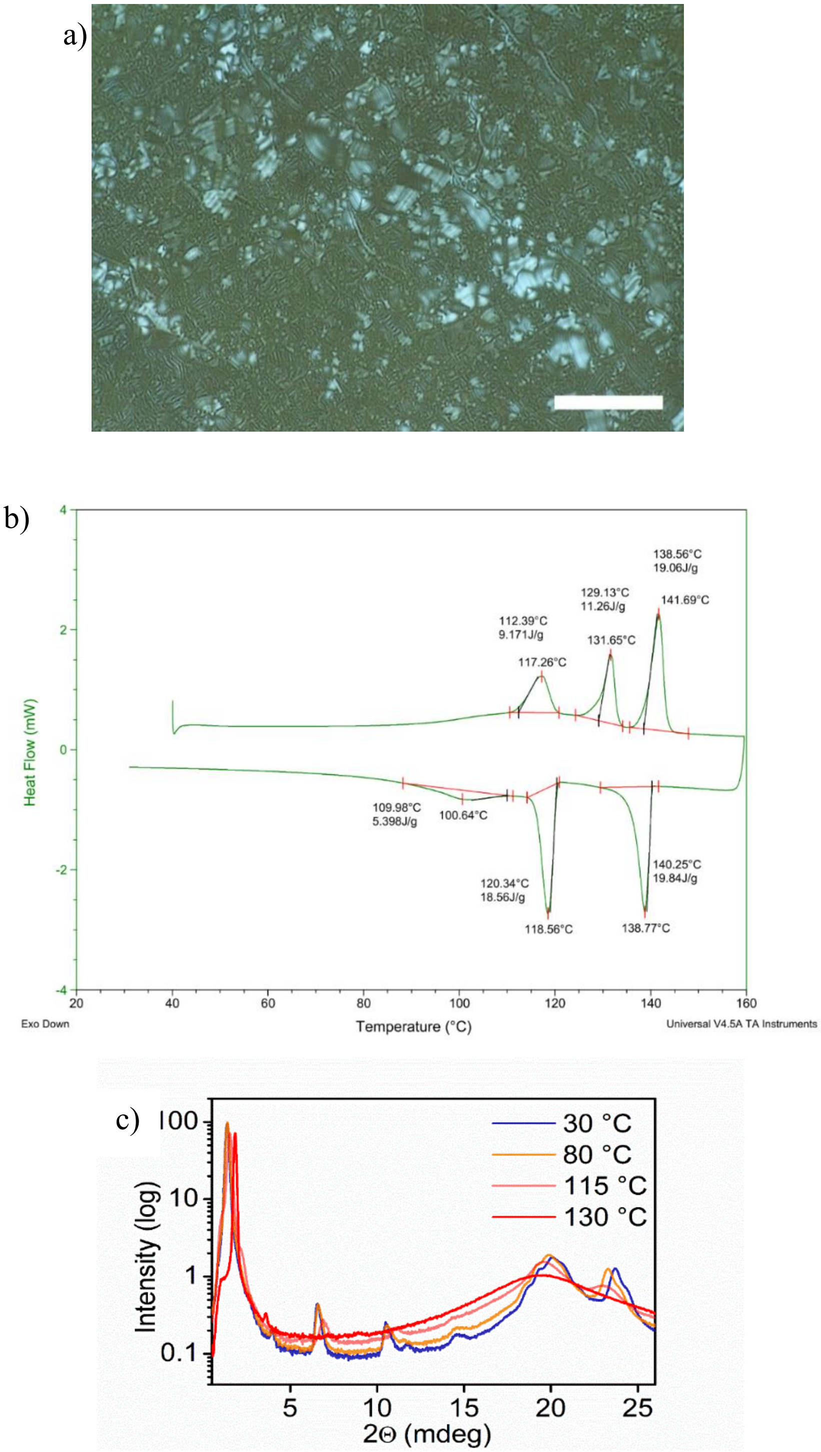


Figure S10. (a) POM image of OIM at an ambient temperature after thermal annealing. The scale bar corresponds to 200 μm. (b) DSC (c) The General Area Detector Diffraction System (GADDS) patterns for OIM in 30, 80, 115, and 130°C.

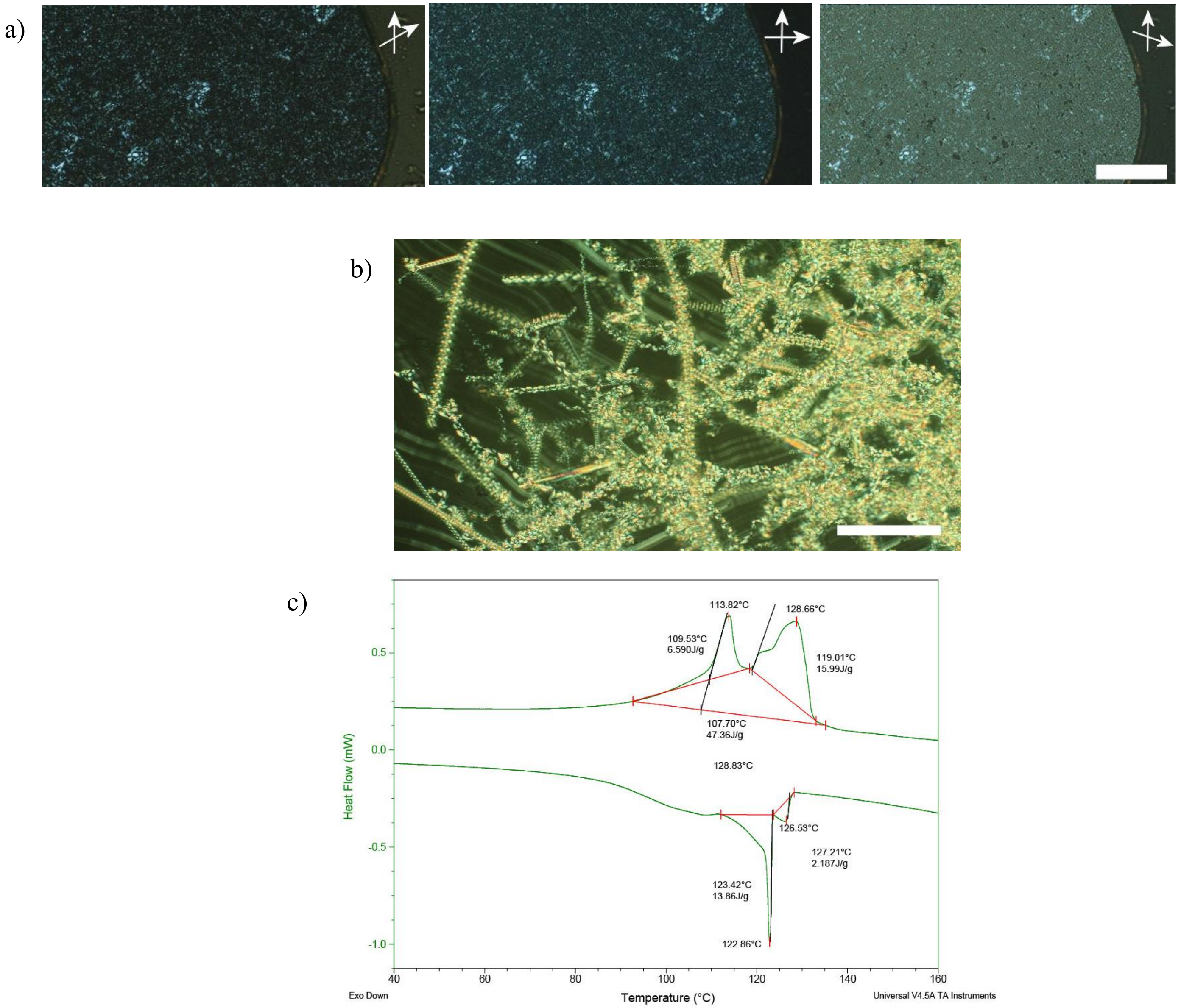


Figure S11. POM images of OIM doped with 5 wt% S5011 ($OIM_S$). (a) At an ambient temperature, showing the optical activity of a single domain; the scale bar corresponds to 100 µm. (b) At 120 °C, showing the texture characteristic to the B7 phase. The scale bar corresponds to 200 µm. (c) Differential scanning calorimetry of $OIM_S$.

a)

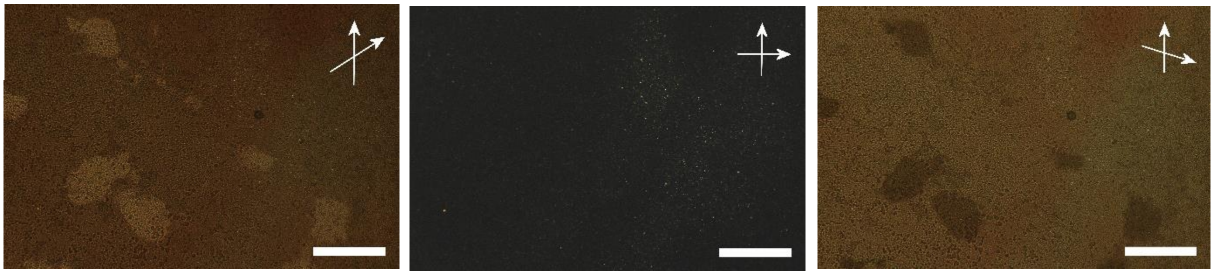

b)

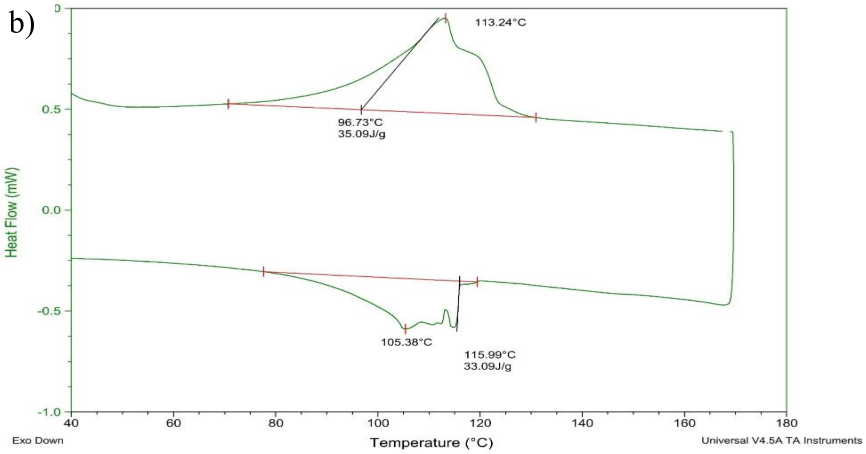


Figure S12. (a) POM images of OIM doped with 5 wt% S5011 and QDs ($OIM_S$_QD4), at an ambient temperature. The scale bar corresponds to 200 μm. (b) Differential Scanning Calorimetry of the OIM doped with 5% S5011 chiral dopant and InP/ZnS QDs ($OIM_S$_QD4).

## Supplementary note 4: Physicochemical characteristics of OIM compound forming helical nanofilaments (1,3-phenylenebis[4-(4-oleyloxy-phenyliminonetyl)benzoate])

Below we present analysis of the mesoscopic structure (particularly the growth of helical nanofilaments on different substrates), as well as analysis of the photoluminescent and chiroptical properties of OIM compound forming helical nanofilaments (1,3-phenylenebis[4-(4-oleyloxy-phenyliminonetyl)benzoate]).

The phenomena of aggregation-induced emission (AIE) in compounds forming helical nanofilaments. To investigate the AIE phenomena we examined two analogous compounds: (1) OIM with two oleyl chains (unsaturated) and (2) P-8-OPIMB with 8 carbon-long, saturated alkyl chains. We drop-casted these materials onto glass substrates, heat-annealed, and measured photoluminescence (Figure S16). For each compound, three samples were prepared:

- fast thermal annealing (25°C/min from the isotropic phase to room temperature)
- slow thermal annealing (3°C/min from the isotropic phase to room temperature)

- chiral doping with slow thermal annealing (3°C/min from the isotropic phase to room temperature).

The above described analysis has proven that both bent-core molecules aggregate into an emissive B4 phase, suggesting that the observed solid-state emission could originate, e.g., from stacking of imine moieties. The presence of oleyl chains seems to improve the brightness of photoluminescence, probably by affecting the aggregation process (Figure S16).

Observations suggest that helical nanofilaments form at different substrates, thus we can correlate ultramicroscopic and bulk spectroscopic analyzes:

- we observe the formation of helical nanofilaments both in direct contact with the substrate, and, in thicker areas, in which the filaments are embedded within the volume of LC material
- in previous works we performed correlated transmission electron microscopy (TEM), atomic force microscopy (AFM), and acanning electron microscopy (SEM) imaging of helical nanofilaments decorated with Au nanoparticles on a TEM grid, ITO-coated glass, and silica substrates; all these results suggested the formation of the same type of structure, with horizontally developed helical nanofilaments of the same pitch and width,
- the appearance of domains of helical nanofilaments on the TEM grid corresponds very well to the mode of HNF phase formation; namely, helical nanofilaments grow dendritically from a crystallization point, preserving handedness;[12,21] this mode of growth, is in line with the formation of conglomerate films by pure OIM which we observe on a glass substrate.

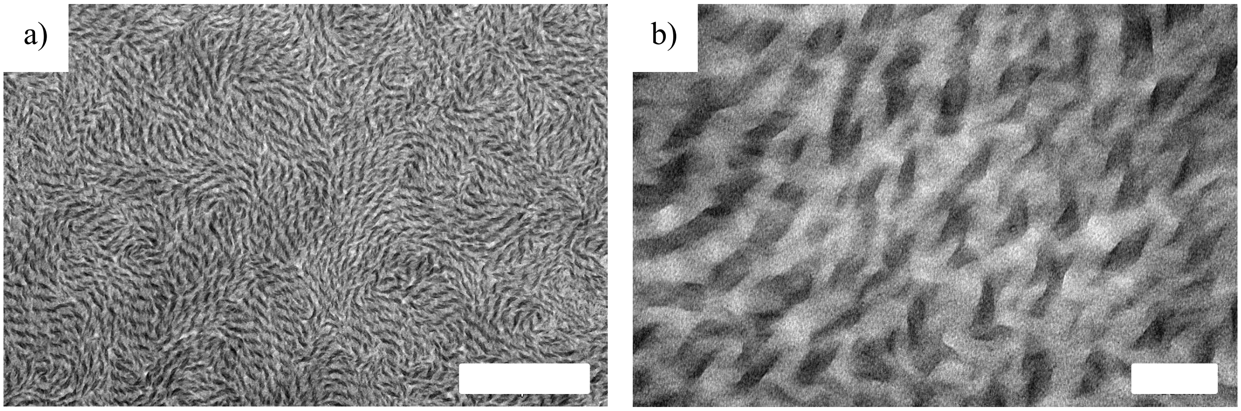


Figure S13. TEM images of thin film of OIM. Densely packed helical nanofilaments are phase-shifted in relation to the nearest neighbor, thus, these micrographs reveal patterns characteristic to bundles of helical nanofilaments. The scale bars correspond to 1 μm (a) and 100 nm (b).

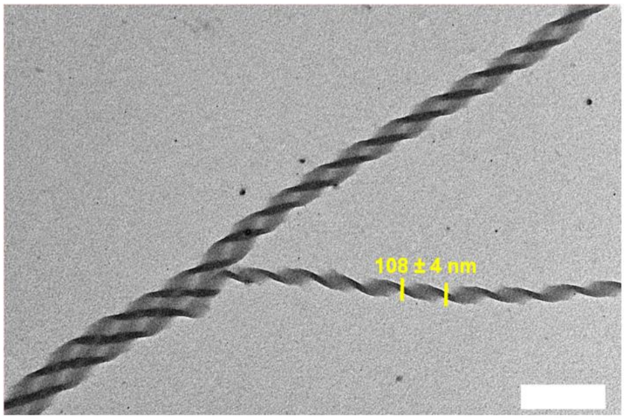


Figure S14. Transmission electron microscopy image of OIM. The average half-pitch of helical nanofilament is 108 ± 4 nm; an exemplary half-pitch used for the calculation is indicated by yellow lines. The scale bar corresponds to 200 nm.

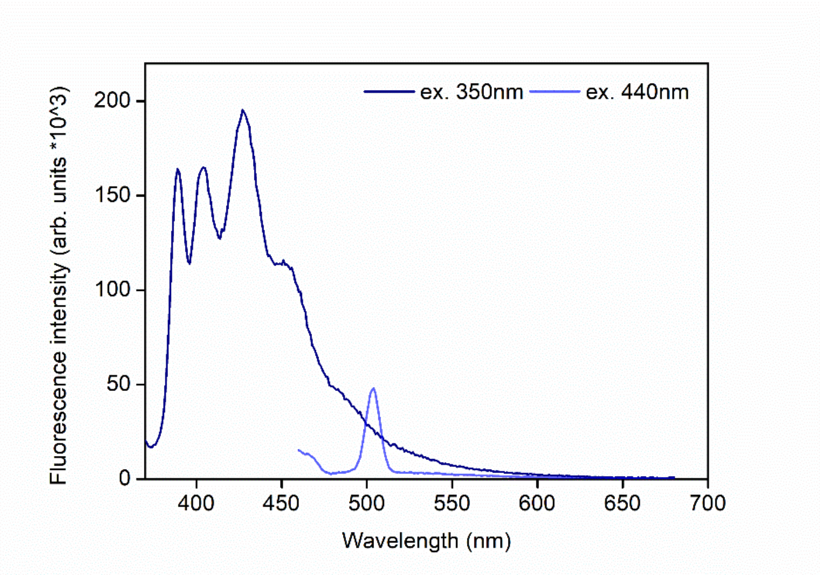


Figure S15. Photoluminescence (PL) of a diluted THF solution of OIM (0.1 mg/ml). Under 350 nm excitation, a weak, broad fluorescence overlapping with strong scattering bands can be observed. Under 440 nm excitation, the solution of OIM exhibits only scattering from the solvent, no OIM emission can be observed.

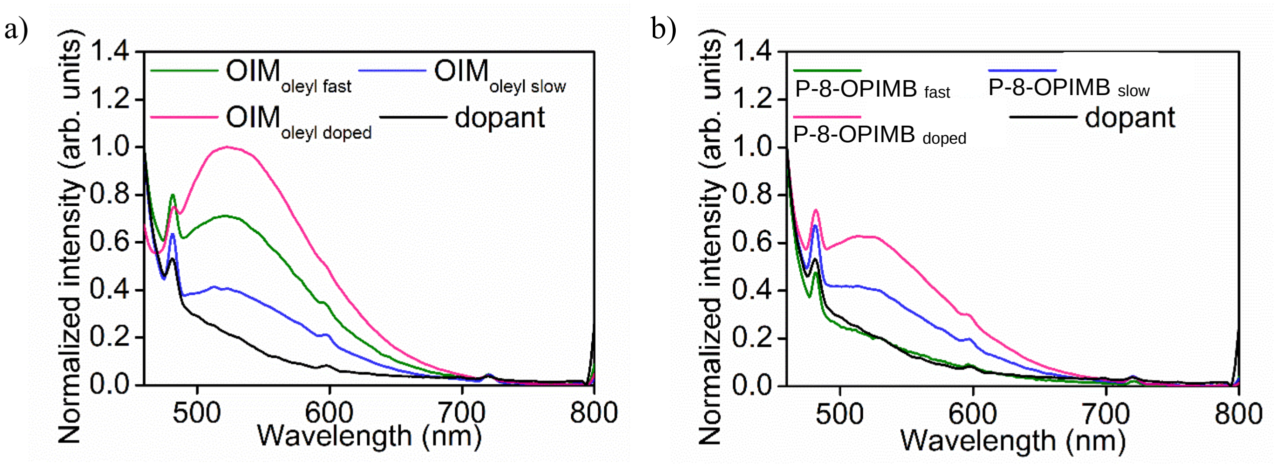


Figure S16. Photoluminescence (PL) spectra of OIM and P-8-OPIMB compounds forming helical nanofilaments. (a) OIM and (b) an analog of OIM having an eight carbon long, saturated terminal alkyl chains. For both compounds, scattering is increasing the spectra intensity in the blue region.

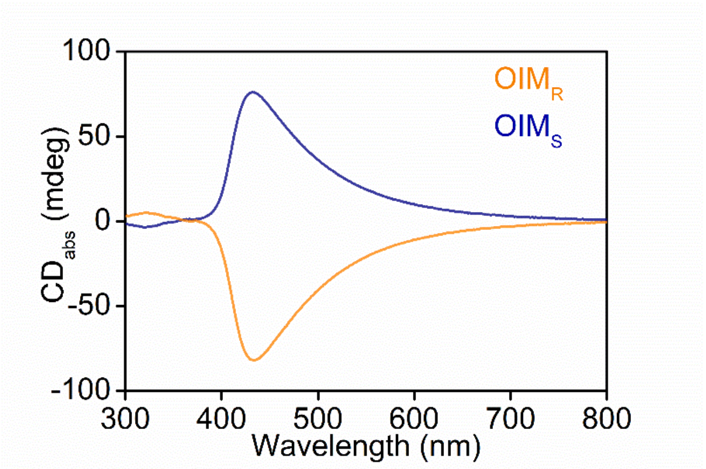


Figure S17. Circular dichroism (CD) spectra of OIM films doped with 5 wt% of R5011 or S5011 (samples $OIM_R$ and $OIM_S$, respectively). The addition of a small amount of a chiral dopant enables achieving an excess of helical nanofilaments of a given handedness.

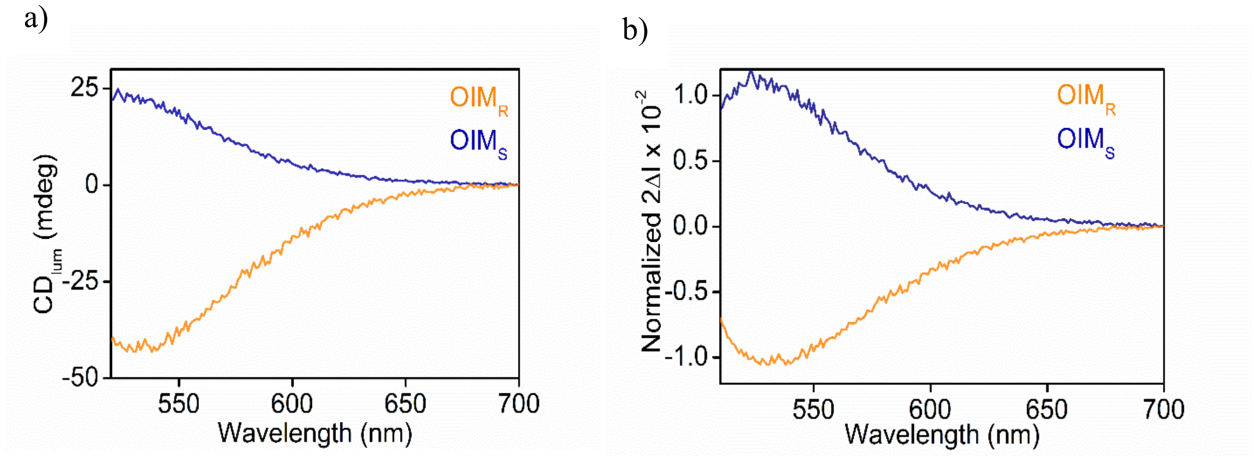


Figure S18. Circularly Polarized Luminescence (CPL) of OIM films doped with 5 wt% of R5011 or S5011 (samples $OIM_R$ and $OIM_S$, respectively). (a) Ellipticity. (b) 2ΔI spectrum, normalized on the maximum of I (λ = 538 nm).

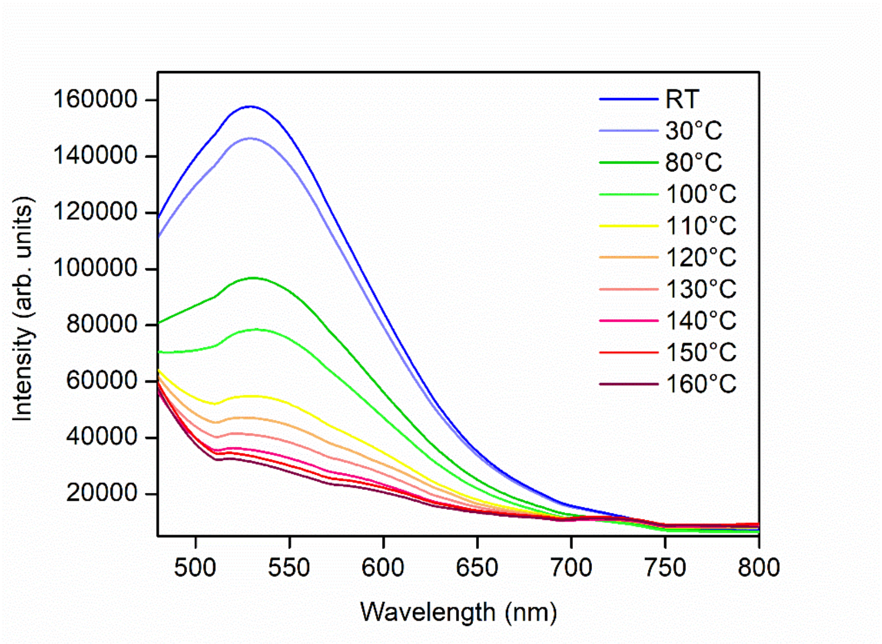


Figure S19. The fluorescence intensity of OIM doped with 5 wt% S5011 ($OIM_S$) at different temperatures. At 110°C the fluorescence intensity drops to ~35% of the initial intensity. AIE liquid crystals offer a fascinating opportunity to create materials with tunable fluorescence properties.[22] Few studies on temperature-dependent, or, in other words, phase-dependent PL/CPL phenomena have already been reported for achiral[23] and chiral AIE LC systems[24]. Notably, in the case of CPL-active AIE LCs increasing the temperature lowers dissymmetry and intensity of emission, however, it seems plausible to assume that further progress in this research area will bring fascinating discoveries and allow for advancing our understanding of CPL/PL of AIE emitters.

## Supplementary note 5: Spectra overlap function, chirality induction

In the case of assemblies of nonchiral building blocks on/within the chiral templates two phrases are often used: chirality induction (or induction of chiroptical properties) and chirality transfer. Following the explanation of chirality transfer phenomena in the terms of coupling transition electric dipole at one component and a magnetic transition dipole at the other, proposed by Andrews,[25] in the case of assemblies of nonchiral building blocks on/within the chiral templates we thus use the term chirality induction or induction of chiroptical properties.[26–30]

To establish the overlap of OIM film emission spectra and QDs absorption, the spectral overlap function was calculated, using the following equation[31] :

$$J(\lambda) = PL_{norm}(\lambda) * \lambda^4 * \varepsilon_{acc}(\lambda)$$

$J(\lambda)$- spectral overlap function [$cm^3/M$]

$PL_{norm}(\lambda)$ - photoluminescence intensity of donor normalized to the area

$\lambda^4$ – wavelength [$cm^4$]

$\varepsilon_{acc}(\lambda)$ – molar extinction coefficient [$M^{-1}cm^{-1}$]

To estimate the molar concentration of QDs in dispersion, required for preparing composite films, UV-Vis spectra and thermogravimetric analysis (TGA) were performed. TGA measurements revealed 35.5 wt% organic content in samples of quantum dots covered only with dodecanethiol ligands (before introducing liquid crystal-like ligand L to the organic shell). This result suggests there are ~39 dodecanethiol molecules per a single nanocrystal (Figure S20). This estimation allowed determining the molar concentration of QDs in dispersion and calculating ε for the dispersion. In the next step, donor's emission was normalized to area 1 and the spectra overlap function was plotted (Figure S21).

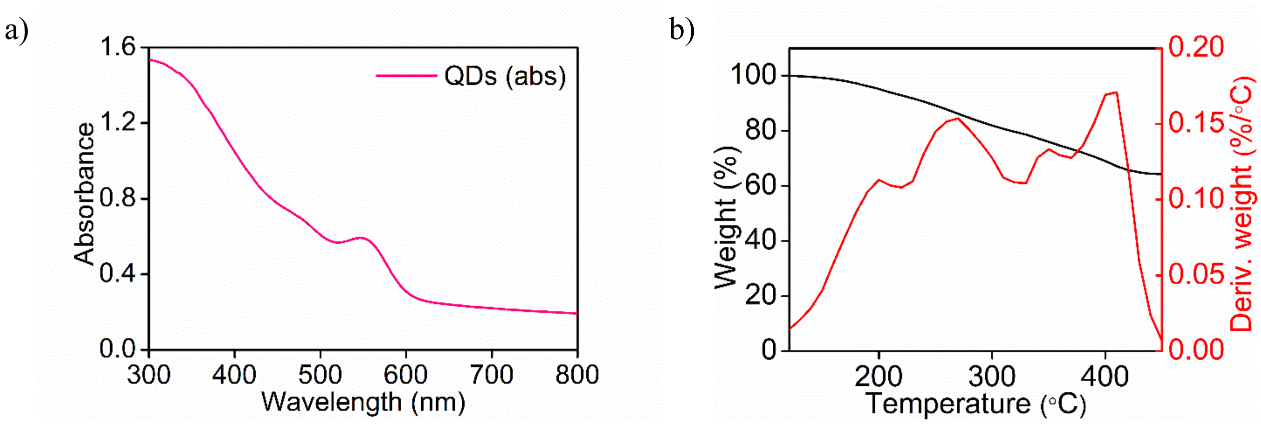


Figure S20. (a) UV- Vis spectra of InP/ZnS quantum dots dispersion in THF. (b) Thermogravimetric (TGA) analysis of dodecanethiol coated InP/ZnS quantum dots. Inorganic content is 64.5%.

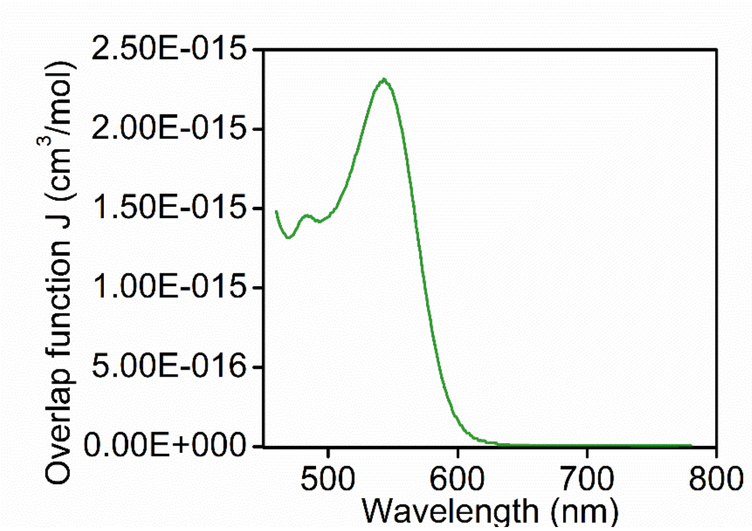


Figure S21. Spectra overlap function, defining the relation between OIMS emission and absorption of InP/ZnS QDs.

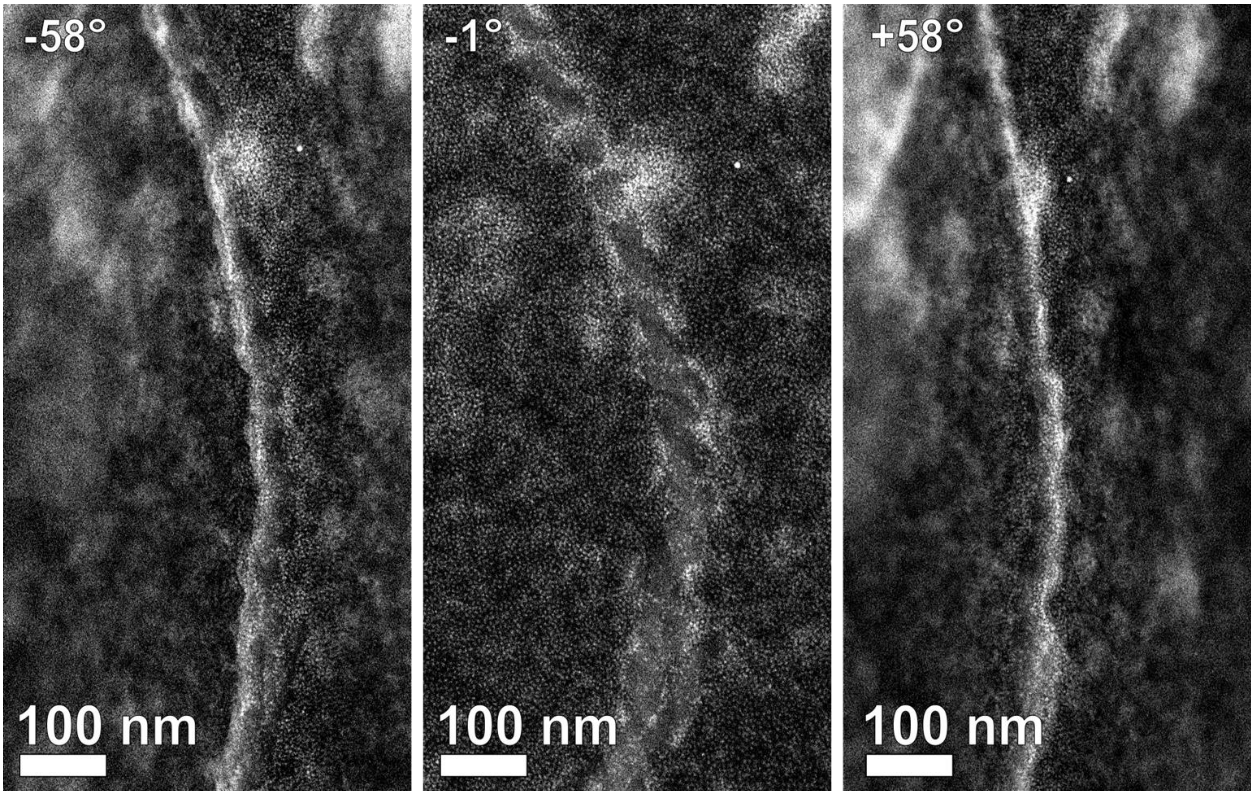


Figure S22. HAADF-STEM (high-angle annular dark-field scanning transmission electron microscopy) images of OIM_QD4 at different tilt angles during the acquisition of a complete tomographic series. QDs (brightest dots) with a helical arrangement on the surface of the organic imine matrix (OIM) filament.

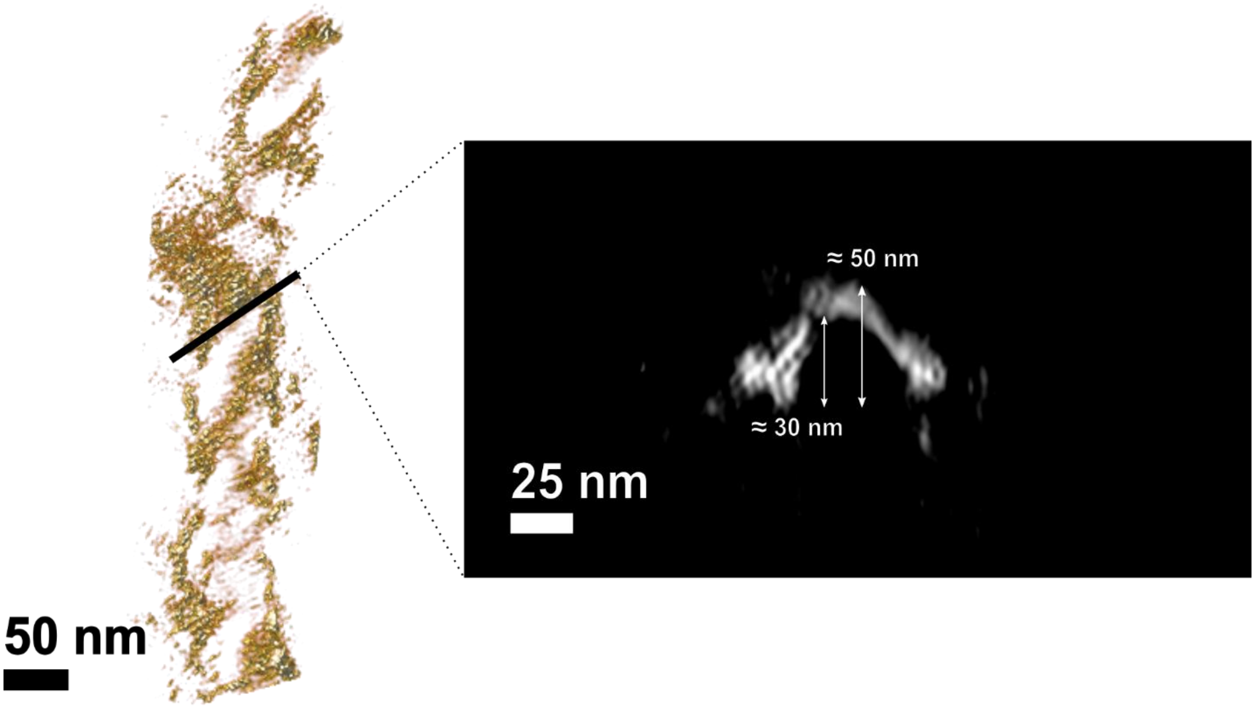


Figure S23. 3D reconstruction (left) and orthoslice (right) from a section of the OIM_QD4 helical filament shown in Figure S21. The position of the orthoslice is indicated in the reconstruction. The height measured from the transversal z-axis indicates a height of 30 nm from the surface of the TEM grid to the bottom edge of the organic structure and 50 nm to the top; these values correspond well to the expected height of ~40 nm (that is the width of the helix).

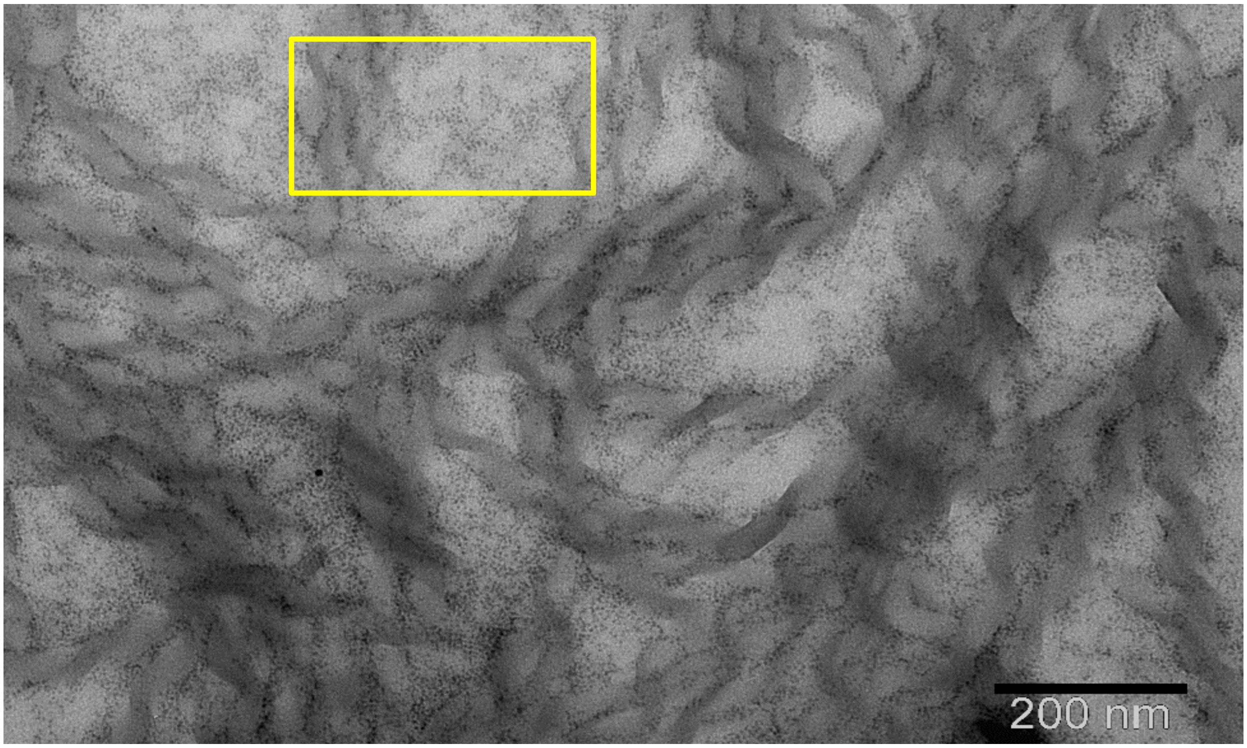


Figure S24. Bright-field transmission electron microscopy (BF-TEM) image of helical nanofilaments covered with QDs (OIM_QD4). An excess of QDs is visible around helical nanofilaments, not deposited onto helices. These QDs contribute to absorbance and fluorescence, but not to CD or CPL, lowering the dissymmetry factors.

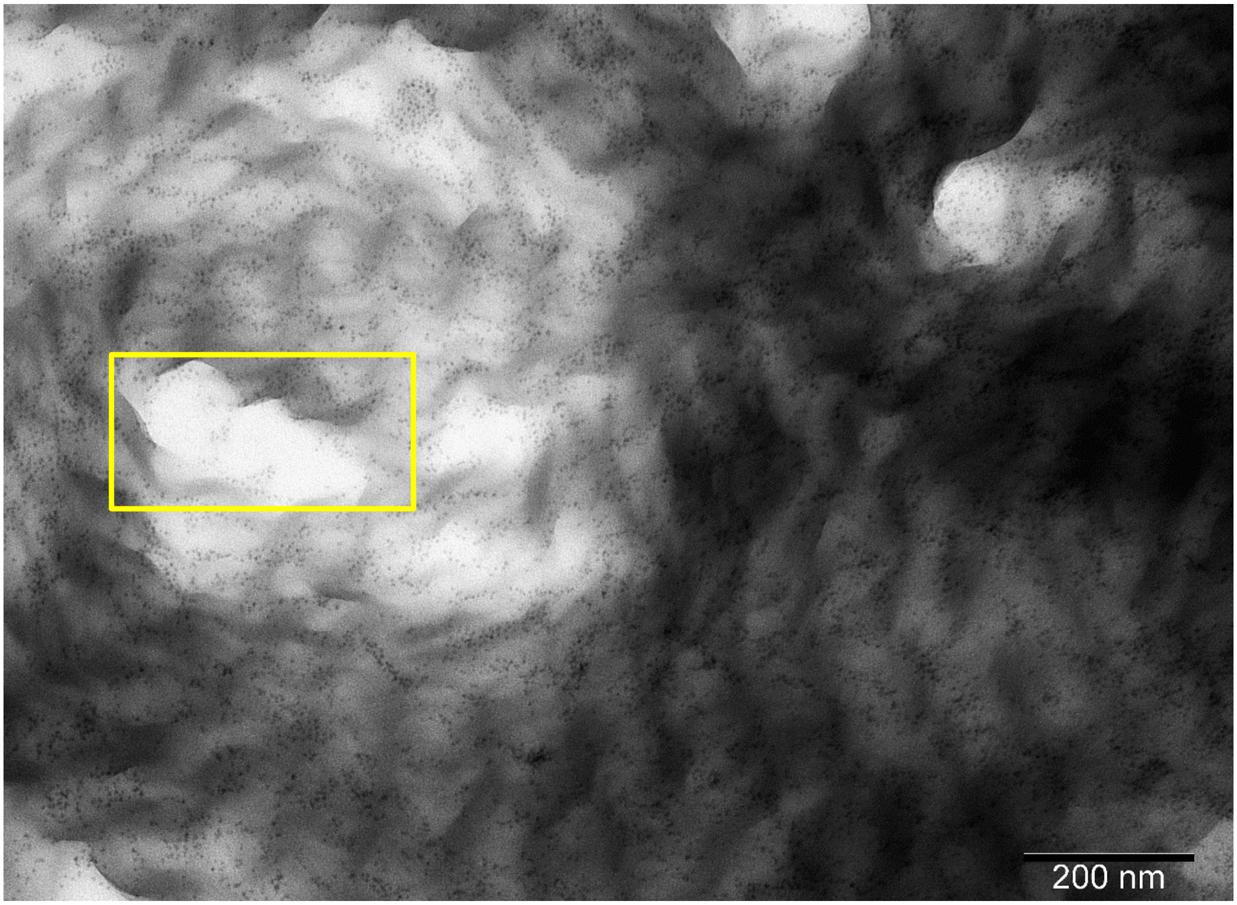


Figure S25. Bright-field transmission electron microscopy (BF-TEM) image of helical nanofilaments covered with QDs (OIM_QD2). The QDs on helices assemble into twisted layers.

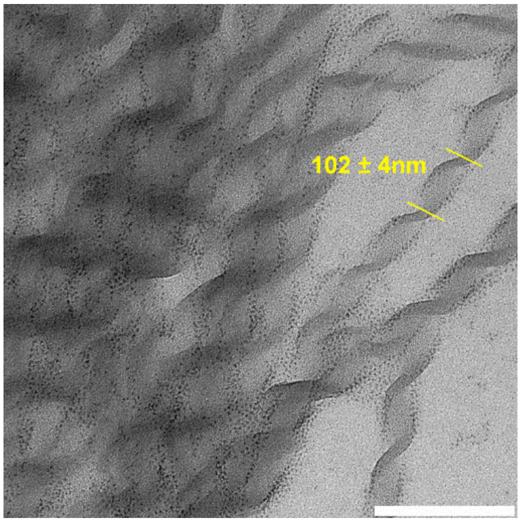


Figure S26. Bright-field transmission electron microscopy (BF-TEM) image of OIM decorated with InP/ZnS QDs (OIM_QD2). The half pitch of helical nanofilament is ~102 nm. The scale bar is 200 nm.

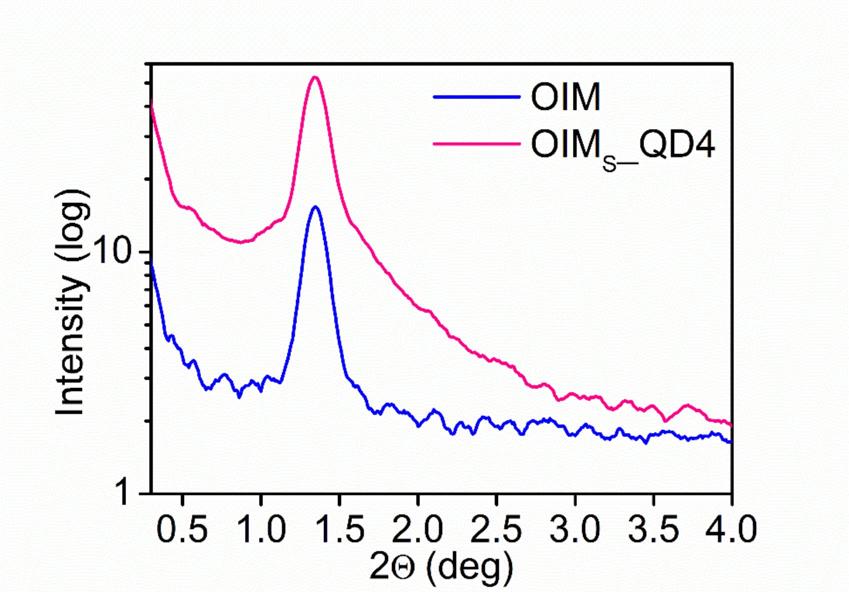


Figure S27. Small angle X-ray diffraction (SAXRD) patterns of thin films: OIM and OIM$_S$_QD4. The presence of QDs in composites is mansifested by an increased scattering around the direct beam (at low angles).

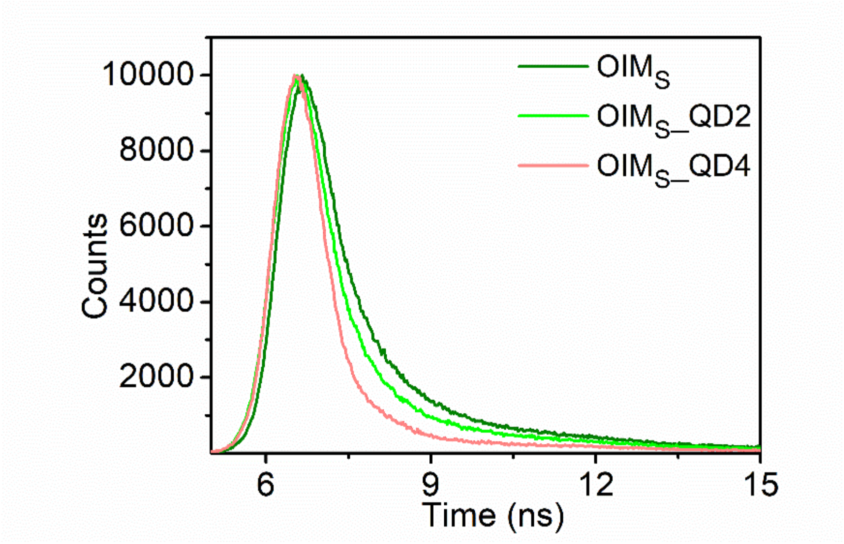


Figure S28. Fluorescence lifetime decay curves of thin films of $OIM_S$ and $OIM_S$-based composites with quantum dots. Decays were measured for the OIM fluorescence band. The addition of QDs results in shortening of the $OIM_S$ PL lifetime, indicating the energy transfer to QDs.

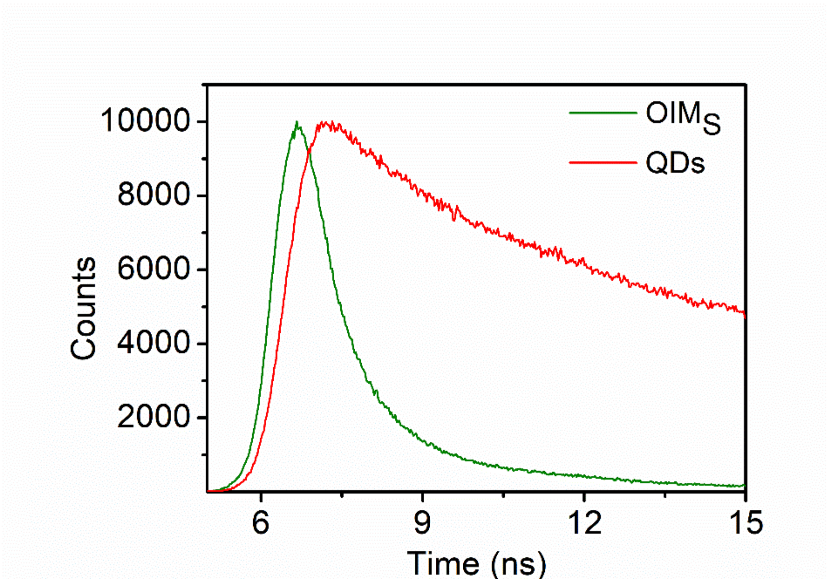


Figure S29. PL lifetime decay curves of thin films of QDs and $OIM_S$. QDs exhibit a longer PL lifetime (hundreds of ns) than $OIM_S$, which is a prerequisite for energy transfer and strenghtenes the energy donor role of $OIM_S$ in the system.

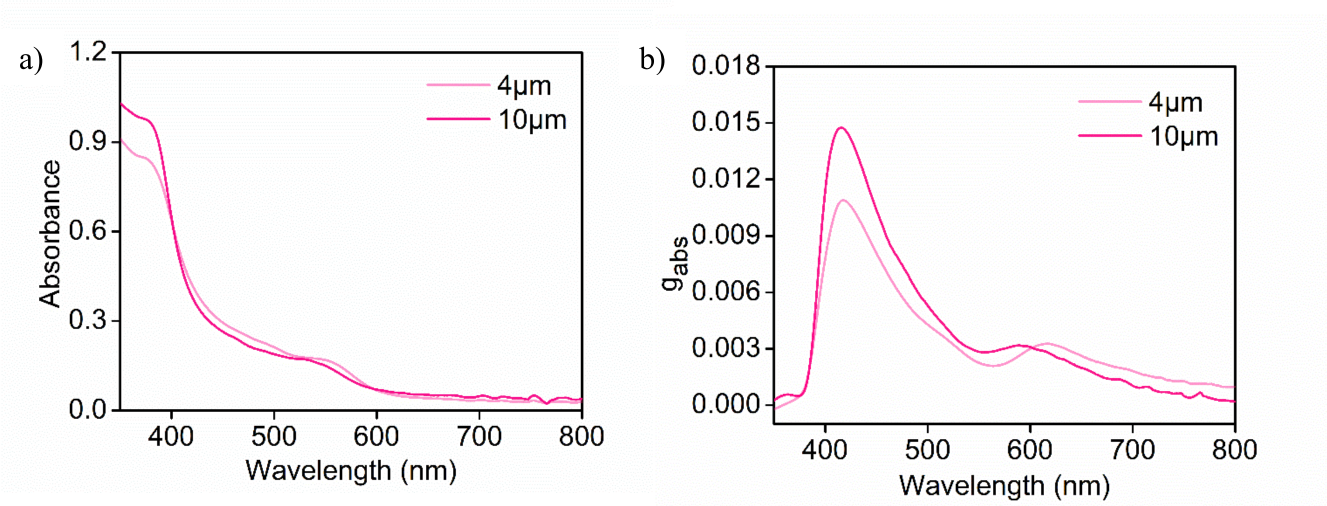


Figure S30. (a) UV-Vis spectra and (b) CD dissymmetry factor of helical nanofilaments decorated with QDs ($OIM_S$_QD4). Each sample was prepared between two coverslips and with 4 or 10 μm spacers.

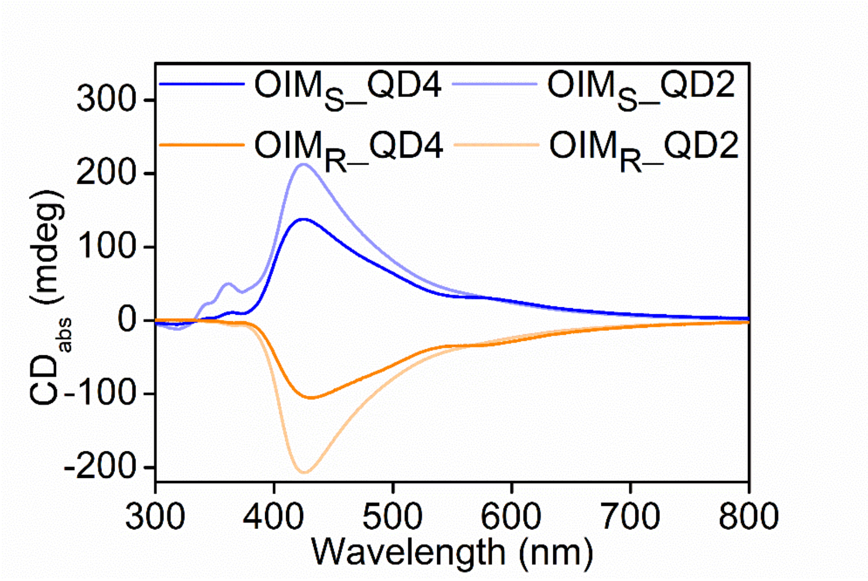


Figure S31. CD ellipticity of composite films based on OIM.

Table S1. CD dissymmetry factor (|$g_{abs}$|) absolute values and the position of the main component of the CD signal (which is characteristic to helical nanofilaments).

| **Sample name** | $OIM_R$ | $OIM_S$ | $OIM_R$_QD4 | $OIM_R$_QD2 | $OIM_S$_QD4 | $OIM_S$_QD2 |
|---|---|---|---|---|---|---|
| Position (nm) | ~439 | ~439 | ~432 | ~435 | ~437 | ~435 |
| $\lvert g_{abs} \rvert$ value | 0.017 | 0.016 | 0.008 | 0.016 | 0.007 | 0.014 |

a) b)

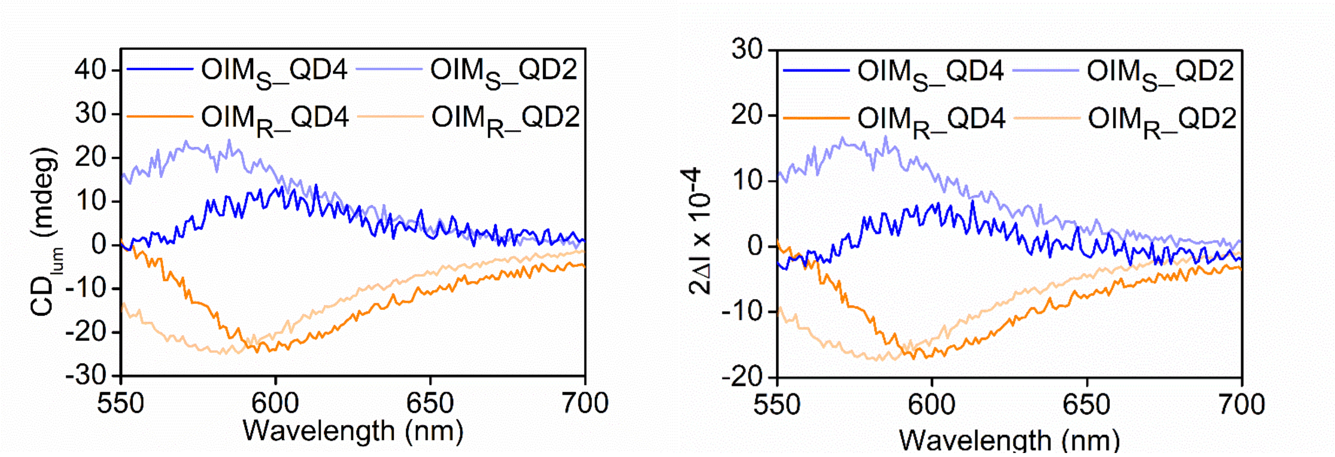


Figure S32. CPL ellipticity of composite films based on OIM. (a) Ellipticity. (b) 2ΔI spectrum, normalized on the maximum of I (λ = 576 nm for $OIM_{R/}S$_QD2 and λ = 597 nm for $OIM_{R/}S$_QD4).

Table S2. Estimated CPL dissymmetry factor (|$g_{lum}$|) absolute values and position of the signal maxima.

| **Sample name** | $OIM_R$ | $OIM_S$ | $OIM_R$_QD4 | $OIM_R$_QD2 | $OIM_S$_QD4 | $OIM_S$_QD2 |
|---|---|---|---|---|---|---|
| Position (nm) | ~538 | ~538 | ~620 | ~590 | ~617 | ~588 |
| $\lvert g_{lum} \rvert$ value | 0.01 | 0.013 | 0.006 | 0.01 | 0.002 | 0.005 |

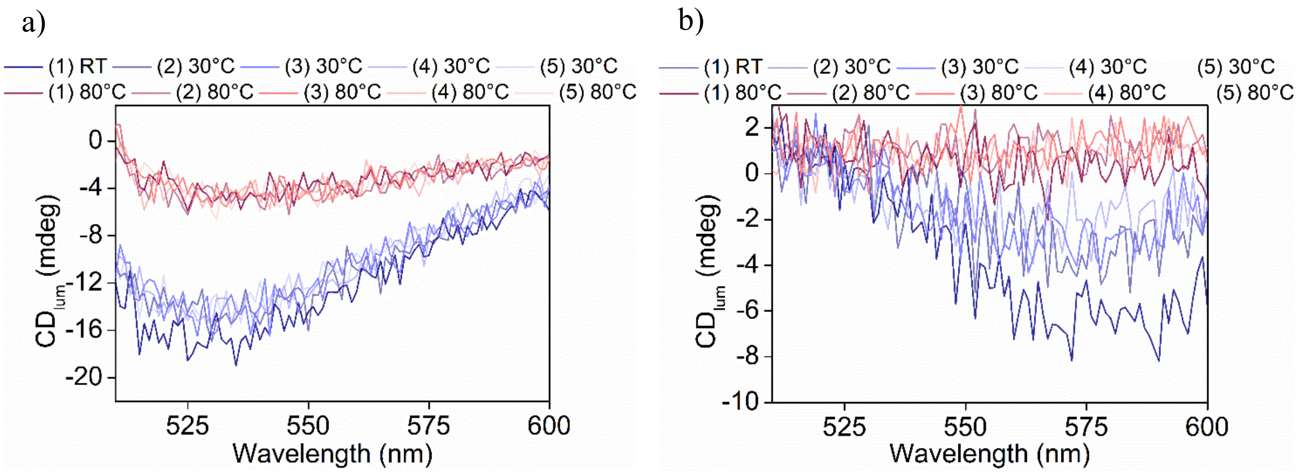


Figure S33. CPL ellipticity of (a) $OIM_S$, (b) helical nanofilaments decorated with QDs ($OIM_S$_QD4).

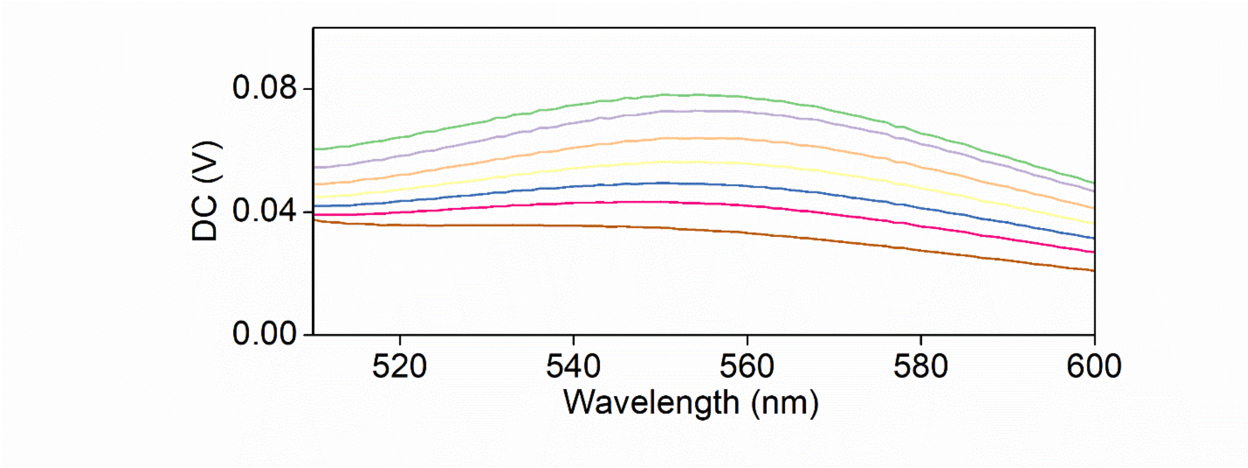


Figure S34. CPL intensity changes with the gradual change of temperature from room temperature to 80 °C of $OIM_S$_QD4.

# Bibliography

(1) Białecka-Florjańczyk, E.; Śledzińska, I.; Górecka, E.; Przedmojski, J. Odd-Even Effect in Biphenyl-Based Symmetrical Dimers with Methylene Spacer - Evidence of the B4 Phase. *Liq Cryst* **2008**, *35*, 401–406.

(2) Cruickshank, E.; Anderson, K.; Storey, J. M. D.; Imrie, C. T.; Gorecka, E.; Pociecha, D.; Makal, A.; Majewska, M. M. Helical Phases Assembled from Achiral Molecules: Twist-Bend Nematic and Helical Filamentary B4 Phases Formed by Mesogenic Dimers. *J Mol Liq* **2022**, *346*, 118180.

(3) Park, W.; Yang, M.; Park, H.; Wolska, J. M.; Ahn, H.; Shin, T. J.; Pociecha, D.; Gorecka, E.; Yoon, D. K. Directing Polymorphism in the Helical Nanofilament Phase. *Chemistry - A European Journal* **2021**, *27,* 7108–7113.

(4) Prasad, V. Bent-Core Mesogens with Biphenyl Moieties: Observation of a B7 to B4 Phase Transition. *Liq Cryst* **2001**, *28,* 1115–1120.

(5) Shadpour, S.; Nemati, A.; Salamończyk, M.; Prévôt, M. E.; Liu, J.; Boyd, N. J.; Wilson, M. R.; Zhu, C.; Hegmann, E.; Jákli, A. I.; Hegmann, T. Missing Link between Helical Nano- and Microfilaments in B4 Phase Bent-Core Liquid Crystals, and Deciphering Which Chiral Center Controls the Filament Handedness. *Small* **2020**, *16*, 1905591.

(6) Liu, J.; Shadpour, S.; Nemati, A.; Prévôt, M. E.; Hegmann, E.; Zhu, C.; Hegmann, T. Binary Mixtures of Bent-Core Molecules Forming Distinct Types of B4 Phase Nano- and Microfilament Morphologies. *Liq Cryst* **2020**, 1129-1139.

(7) Park, W.; Yoon, D. K. Orientation Control of Helical Nanofilament Phase and Its Chiroptical Applications. *Crystals* **2020**, *10*, 675.

(8) Bagiński, M.; Tupikowska, M.; González-Rubio, G.; Wójcik, M.; Lewandowski, W. Shaping Liquid Crystals with Gold Nanoparticles: Helical Assemblies with Tunable and Hierarchical Structures Via Thin-Film Cooperative Interactions. *Adv. Mater.* **2020**, *32*, 1904581.

(9) Liu, J.; Shadpour, S.; Prévôt, M. E.; Chirgwin, M.; Nemati, A.; Hegmann, E.; Lemieux, R. P.; Hegmann, T. Molecular Conformation of Bent-Core Molecules Affected by Chiral Side Chains Dictates Polymorphism and Chirality in Organic Nano- And Microfilaments. *ACS Nano* **2021**, *15*, 7249–7270.

(10) Li, L.; Salamonczyk, M.; Jákli, A.; Hegmann, T. A Dual Modulated Homochiral Helical Nanofilament Phase with Local Columnar Ordering Formed by Bent Core Liquid Crystals: Effects of Molecular Chirality. *Small* **2016**, *12,* 3944–3955.

(11) Thisayukta, J.; Niwano, H.; Takezoe, H.; Watanabe, J. Effect of Chiral Dopant on a Helical Sm1 Phase of Banana-Shaped N-n-O-PIMB Molecules. *J Mater Chem* **2001**, *11*, 2717–2721.

(12) Grzelak, D.; Tupikowska, M.; Vila-Liarte, D.; Beutel, D.; Bagiński, M.; Parzyszek, S.; Góra, M.; Rockstuhl, C.; Liz-Marzán, L. M.; Lewandowski, W. Liquid Crystal Templated Chiral Plasmonic Films with Dynamic Tunability and Moldability. *Adv Funct Mater* **2022**, *32,* 2111280.

(13) Shiromo, K.; Sahade, D. A.; Oda, T.; Nihira, T.; Takanishi, Y.; Ishikawa, K.; Takezoe, H. Finite Enantiomeric Excess Nucleated in an Achiral Banana Mesogen by Chiral Alignment Surfaces. *Angew. Chem. Int. Ed.* **2005**, *44*, 1948–1951.

(14) Lee, G.; Carlton, R. J.; Araoka, F.; Abbott, N. L.; Takezoe, H. Amplification of the Stereochemistry of Biomolecular Adsorbates by Deracemization of Chiral Domains in Bent-Core Liquid Crystals. *Adv Mater* **2013**, *25*, 245–249.

(15) Choi, S.-W.; Izumi, T.; Hoshino, Y.; Takanishi, Y.; Ishikawa, K.; Watanabe, J.; Takezoe, H. Circular-Polarization-Induced Enantiomeric Excess in Liquid Crystals of an Achiral, Bent-Shaped Mesogen. *Angew. Chem. Int. Ed.* **2006**, *45*, 1382–1385.

(16) Hoshino, Y.; Choi, S.-W.; Izumi, T.; Takanishi, Y.; Ishikawa, K.; Watanabe, J.; Takezoe, H. Chirality Induced by Circularly Polarized Light in Liquid Crystalline Twin Dimers with Azo Linkages. *Molecular Crystals and Liquid Crystals* **2007**, *465*, 153–163.

(17) Choi, S.-W.; Kang, S.; Takanishi, Y.; Ishikawa, K.; Watanabe, J.; Takezoe, H. Intrinsic Chirality in a Bent-Core Mesogen Induced by Extrinsic Chiral Structures. *Angew. Chem. Int. Ed.* **2006**, *45*, 6503–6506.

(18) Lee, D.-M.; Song, J.-W.; Lee, Y.-J.; Yu, C.-J.; Kim, J.-H. Control of Circularly Polarized Electroluminescence in Induced Twist Structure of Conjugate Polymer. *Adv. Mater.* **2017**, *29*, 1700907.

(19) Li, Z.; Lan, R.; Bao, J.; Hu, W.; Wang, M.; Zhang, L.; Yang, H. Tunable Circularly Polarized Luminescence with a High Dissymmetry Factor Emitted from Luminogen-Bonded and Electrically Controlled Polymer-Stabilized Cholesteric Liquid Crystals. *ACS Appl Mater Interfaces* **2022**, *14*, 8490–8498.

(20) Hough, L. E.; Jung, H. T.; Krüerke, D.; Heberling, M. S.; Nakata, M.; Jones, C. D.; Chen, D.; Link, D. R.; Zasadzinski, J.; Heppke, G.; Rabe, J. P.; Stocker, W.; Körblova, E.; Walba, D. M.; Glaser, M. A.; Clark, N. A. Helical Nanofilament Phases. *Science* **2009**, *325* (5939), 456–460.

(21) Lewandowski, W.; Vaupotič, N.; Pociecha, D.; Górecka, E.; Liz-Marzán, L. M. Chirality of Liquid Crystals Formed from Achiral Molecules Revealed by Resonant X-Ray Scattering. *Adv. Mater.* **2020**, *32*, 1905591.

(22) Voskuhl, J.; Giese, M. Mesogens with Aggregation-induced Emission Properties: Materials with a Bright Future. *Aggregate* **2022**, *3* (1), e124.

(23) Kim, J.; Cho, S.; Cho, B.-K. An Unusual Stacking Transformation in Liquid-Crystalline Columnar Assemblies of Clicked Molecular Propellers with Tunable Light Emissions. *Chemistry - A European Journal* **2014**, *20*, 12734–12739.

(24) Jiang, S.; Qiu, J.; Lin, L.; Guo, H.; Yang, F. Circularly Polarized Luminescence Based on Columnar Self-Assembly of Tetraphenylethylene with Multiple Cholesterol Units. *Dyes and Pigments* **2019**, *163*, 363–370.

(25) Andrews, D. L. Chirality in Fluorescence and Energy Transfer. *Methods Appl Fluoresc* **2019**, *7*, 32001.

(26) Wade, J.; Brandt, J. R.; Reger, D.; Zinna, F.; Amsharov, K. Y.; Jux, N.; Andrews, D. L.; Fuchter, M. J. 500-Fold Amplification of Small Molecule Circularly Polarised Luminescence through Circularly Polarised FRET. *Angew. Chem. Int. Ed.* **2021**, *60*, 222–227.

(27) Li, Y.; Duan, P.; Liu, M. Solvent-Regulated Self-Assembly of an Achiral Donor-Acceptor Complex in Confined Chiral Nanotubes: Chirality Transfer, Inversion and Amplification. *Chemistry - A European Journal* **2017**, *23*, 8225–8231.

(28) Yang, D.; Duan, P.; Zhang, L.; Liu, M. Chirality and Energy Transfer Amplified Circularly Polarized Luminescence in Composite Nanohelix. *Nat Commun* **2017**, *8*, 15727.

(29) Wen, X.; Fan, H.; Jing, L.; Deng, M.; Huang, X.; Jiao, T.; Zhang, L.; Liu, M. Competitive Induction of Circularly Polarized Luminescence of CdSe/ZnS Quantum Dots in a Nucleotide–Amino Acid Hydrogel. *Mater Adv* **2022**, *3*, 682–688.

(30) Huo, S.; Duan, P.; Jiao, T.; Peng, Q.; Liu, M. Self-Assembled Luminescent Quantum Dots To Generate Full-Color and White Circularly Polarized Light. *Angew. Chem. Int. Ed.* **2017**, *56*, 12174–12178.

(31) Clapp, A. R.; Medintz, I. L.; Fisher, B. R.; Anderson, G. P.; Mattoussi, H. Can Luminescent Quantum Dots Be Efficient Energy Acceptors with Organic Dye Donors? *J Am Chem Soc* **2005**, *127* (4), 1242–1250.
